# Body Size at Different Ages and Risk of 6 Cancers: A Mendelian Randomization and Prospective Cohort Study

**DOI:** 10.1093/jnci/djac061

**Published:** 2022-04-19

**Authors:** Daniela Mariosa, Karl Smith-Byrne, Tom G Richardson, Pietro Ferrari, Marc J Gunter, Nikos Papadimitriou, Neil Murphy, Sofia Christakoudi, Konstantinos K Tsilidis, Elio Riboli, David Muller, Mark P Purdue, Stephen J Chanock, Rayjean J Hung, Christopher I Amos, Tracy A O’Mara, Pilar Amiano, Fabrizio Pasanisi, Miguel Rodriguez-Barranco, Vittorio Krogh, Anne Tjønneland, Jytte Halkjær, Aurora Perez-Cornago, María-Dolores Chirlaque, Guri Skeie, Charlotta Rylander, Kristin Benjaminsen Borch, Dagfinn Aune, Alicia K Heath, Heather A Ward, Matthias Schulze, Catalina Bonet, Elisabete Weiderpass, George Davey Smith, Paul Brennan, Mattias Johansson

**Affiliations:** International Agency for Research on Cancer (IARC/WHO), Genomic Epidemiology Branch, Lyon, France; International Agency for Research on Cancer (IARC/WHO), Genomic Epidemiology Branch, Lyon, France; MRC Integrative Epidemiology Unit (IEU), Population Health Sciences, Bristol Medical School, University of Bristol, Bristol, UK; Novo Nordisk Research Centre Oxford, Oxford, UK; International Agency for Research on Cancer (IARC/WHO), Nutrition and Metabolism Branch, Lyon, France; International Agency for Research on Cancer (IARC/WHO), Nutrition and Metabolism Branch, Lyon, France; International Agency for Research on Cancer (IARC/WHO), Nutrition and Metabolism Branch, Lyon, France; International Agency for Research on Cancer (IARC/WHO), Nutrition and Metabolism Branch, Lyon, France; Department of Epidemiology and Biostatistics, School of Public Health, Imperial College London, London, UK; MRC Centre for Transplantation, King’s College London, London, UK; Department of Epidemiology and Biostatistics, School of Public Health, Imperial College London, London, UK; Department of Hygiene and Epidemiology, School of Medicine, University of Ioannina, Ioannina, Greece; Department of Epidemiology and Biostatistics, School of Public Health, Imperial College London, London, UK; Department of Epidemiology and Biostatistics, School of Public Health, Imperial College London, London, UK; Division of Cancer Epidemiology and Genetics, National Cancer Institute, Rockville, MD, USA; Division of Cancer Epidemiology and Genetics, National Cancer Institute, Rockville, MD, USA; Prosserman Centre for Population Health Research, Lunenfeld-Tanenbaum Research Institute, Sinai Health System, Toronto, ON, Canada; Division of Epidemiology, Dalla Lana School of Public Health, University of Toronto, Toronto, ON, Canada; Institute for Clinical and Translational Research, Baylor College of Medicine, Houston, TX, USA; Department of Genetics and Computational Biology, QIMR Berghofer Medical Research Institute, Brisbane, Australia; Ministry of Health of the Basque Government, Sub Directorate for Public Health and Addictions of Gipuzkoa, San Sebastián, Spain; Epidemiology of Chronic and Communicable Diseases Group, Biodonostia Health Research Institute, San Sebastián, Spain; Spanish Consortium for Research on Epidemiology and Public Health (CIBERESP), Instituto de Salud Carlos III, Madrid, Spain; Dipartimento di Medicina Clinica e Chirurgia, Federico II University, Naples, Italy; Escuela Andaluza de Salud Pública (EASP), Granada, Spain; Instituto de Investigación Biosanitaria ibs.GRANADA, Granada, Spain; Centro de Investigación Biomédica en Red de Epidemiología y Salud Pública (CIBERESP), Madrid, Spain; Epidemiology and Prevention Unit, Fondazione IRCCS Istituto Nazionale dei Tumori, Milan, Italy; Danish Cancer Society Research Center, Copenhagen, Denmark; Department of Public Health, Faculty of Health and Medical Sciences, University of Copenhagen, Copenhagen, Denmark; Danish Cancer Society Research Center, Copenhagen, Denmark; Cancer Epidemiology Unit, Nuffield Department of Population Health, University of Oxford, Oxford, UK; Centro de Investigación Biomédica en Red de Epidemiología y Salud Pública (CIBERESP), Madrid, Spain; Department of Epidemiology, Regional Health Council, IMIB-Arrixaca, Murcia University, Murcia, Spain; Department of Community Medicine, UiT, The Arctic University of Norway, Tromsø, Norway; Department of Community Medicine, UiT, The Arctic University of Norway, Tromsø, Norway; Department of Community Medicine, UiT, The Arctic University of Norway, Tromsø, Norway; Department of Epidemiology and Biostatistics, School of Public Health, Imperial College London, London, UK; Department of Nutrition, Bjørknes University College, Oslo, Norway; Department of Endocrinology, Morbid Obesity and Preventive Medicine, Oslo University Hospital, Oslo, Norway; Unit of Cardiovascular and Nutritional Epidemiology, Institute of Environmental Medicine, Karolinska Institutet, Stockholm, Sweden; Department of Epidemiology and Biostatistics, School of Public Health, Imperial College London, London, UK; Department of Epidemiology and Biostatistics, School of Public Health, Imperial College London, London, UK; IQVIA, Epidemiology and Outcomes Research, Real World Solutions, IQVIA, Cambridge, MA, USA; Department of Molecular Epidemiology, German Institute of Human Nutrition Potsdam-Rehbruecke, Nuthetal, Germany; Unit of Nutrition and Cancer, Cancer Epidemiology Research Program, Catalan Institute of Oncology- IDIBELL, L’Hospitalet de Llobregat, Barcelona, Spain; International Agency for Research on Cancer (IARC/WHO), Lyon, France; MRC Integrative Epidemiology Unit (IEU), Population Health Sciences, Bristol Medical School, University of Bristol, Bristol, UK; International Agency for Research on Cancer (IARC/WHO), Genomic Epidemiology Branch, Lyon, France; International Agency for Research on Cancer (IARC/WHO), Genomic Epidemiology Branch, Lyon, France

## Abstract

It is unclear if body weight in early life affects cancer risk independently of adult body weight. To investigate this question for 6 obesity-related cancers, we performed univariable and multivariable analyses using 1) Mendelian randomization (MR) analysis and 2) longitudinal analyses in prospective cohorts. Both the MR and longitudinal analyses indicated that larger early life body size was associated with higher risk of endometrial (odds ratio_MR_ = 1.61, 95% confidence interval = 1.23 to 2.11) and kidney (odds ratio_MR_ = 1.40, 95% confidence interval = 1.09 to 1.80) cancer. These associations were attenuated after accounting for adult body size in both the MR and cohort analyses. Early life body mass index (BMI) was not consistently associated with the other investigated cancers. The lack of clear independent risk associations suggests that early life BMI influences endometrial and kidney cancer risk mainly through pathways that are common with adult BMI.

Adult obesity is associated with increased risk of several common cancers (1,2). Body mass index (BMI) in children and young adults is also associated with cancer risk (3-12), but the extent to which body weight in early life affects cancer risk independently of body weight later in life is poorly understood. Mendelian randomization (MR) studies using genetic proxies for BMI have generally confirmed previously reported associations for BMI from large, longitudinal cohort studies (13–19). A recent MR study found that elevated childhood BMI was associated with a decreased risk of breast cancer, whereas adult BMI had no additional effect on risk after accounting for childhood BMI (20). Whether other cancers present a similar pattern is largely unknown.

We sought to investigate body size at different ages in relation to risk of 6 common obesity-related cancers by carrying out 2 complementary lines of analyses using 1) genetic proxies for body size in an MR framework and 2) BMI measurements in large, prospective cohort studies, respectively.

We identified genetic instruments for body size at age 10 years and at ages 40-69 years in 453 169 UK Biobank participants. The instruments were subsequently evaluated in relation to risk of cancer of the colorectum, kidney, pancreas, lung, ovary, and endometrium using summary statistics from genome-wide association studies of between 10 000 and 100 000 samples (Supplementary Methods, available online)(21–28). There was no clear violation of the NO Measurement Error assumption and instruments explained between 2% and 5% of the body size variance (Supplementary Table 1, available online). We estimated odds ratios (ORs) of cancer for genetically predicted body size at age 10 years and adult body size, initially using univariable MR to estimate their main effects and subsequently using multivariable MR to evaluate their independence (29).

In parallel with the MR analysis, we conducted a longitudinal cohort analysis for the association of BMI at ages 18-20 years and 40-69 years with cancer risk in 185 361 participants of the European Prospective Investigation into Cancer and Nutrition study (Supplementary Methods, available online). We estimated hazard ratios of cancer for BMI at age 18-20 years and 40-69 years using Cox proportional hazards regression models and subsequently fitted mutually adjusted models to evaluate their independence (Supplementary Methods, available online). All statistical tests were 2-sided and a *P* less than .05 was considered statistically significant.

We found concordant risk association results in both MR and cohort analyses for kidney and endometrial cancer (Figure 1). Larger body size at age 10 years (OR = 1.40, 95% confidence interval [CI] = 1.09 to 1.80) and adult body size (OR = 1.74, 95% CI = 1.43 to 2.11) were clearly associated with higher kidney cancer risk in univariable MR. Similarly, higher BMI at ages 18-20 years and 40-69 years was associated with higher risk in the corresponding cohort analysis. The risk associations for adult body size remained in mutually adjusted multivariable analyses, whereas the associations for early life body size were attenuated (Figure 1). We found a similar pattern of risk associations for endometrial cancer, with early life (OR = 1.61, 95% CI = 1.23 to 2.11) and adult (OR = 2.19, 95% CI = 1.79 to 2.69) body size clearly associated with risk in univariable MR. The risk association for early life body size was attenuated in multivariable MR but became inverse in the cohort analysis adjusted for adult BMI.

**Figure 1.**
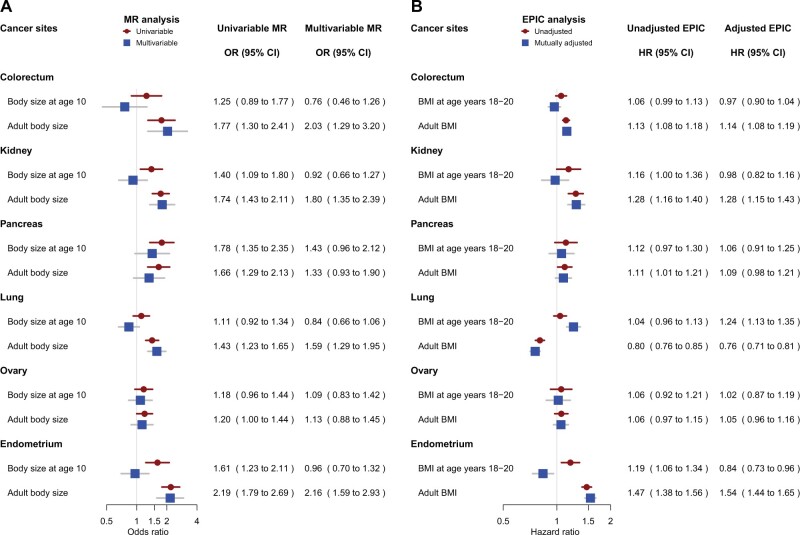
Mendelian randomization (MR) results and EPIC cohort results for different cancer sites. **A)** Odds ratios (ORs) and 95% confidence intervals (CIs) for category increase (ie, thinner than average, average, larger than average) in body size at age 10 years and adult body size before (univariable) and after (multivariable) mutual adjustment. **B)** Hazard ratios (HRs) for a 5-unit increase in BMI expressed in kg/m^2^ at age 18-20 years and in adulthood before (unadjusted) and after (adjusted) mutual adjustment. BMI = body mass index; EPIC = European Prospective Investigation into Cancer and Nutrition.

The associations of body size at different ages with risk of colorectal, pancreatic, lung, and ovarian cancer were less clear (Figure 1). For colorectal cancer, early life body size was not clearly associated with risk neither in MR nor in cohort analyses. For pancreatic cancer, the MR and cohort analyses showed similar risk associations for early life (univariable MR OR = 1.78, 95% CI = 1.35 to 2.35) and adult BMI (univariable MR OR = 1.66, 95% CI = 1.29 to 2.13), but mutually adjusted analyses slightly attenuated the risk association estimates for both exposures. The associations for lung cancer varied by histology (Figure 2). In MR, adult body size was associated with higher lung cancer risk to a various extent for different subtypes, whereas body size at age 10 years was not associated with risk after accounting for adult body size (Figure 2, A). In contrast, adult BMI was inversely associated with risk of lung squamous cell and adenocarcinoma in the cohort analysis, and BMI at age 18-20 years was positively associated with lung cancer risk after mutual adjustment but not after additional adjustment for smoking (Figure 2, B and C). The relationship between BMI and lung cancer risk is complex because obesity and smoking affect each other (30), but the 2 approaches taken together suggest that early life BMI may increase lung cancer risk through its effect on smoking behavior. For ovarian cancer overall, MR and the longitudinal analysis showed weak associations of early life or adult body size with risk (31) (Supplementary Table 2, available online).

**Figure 2.**
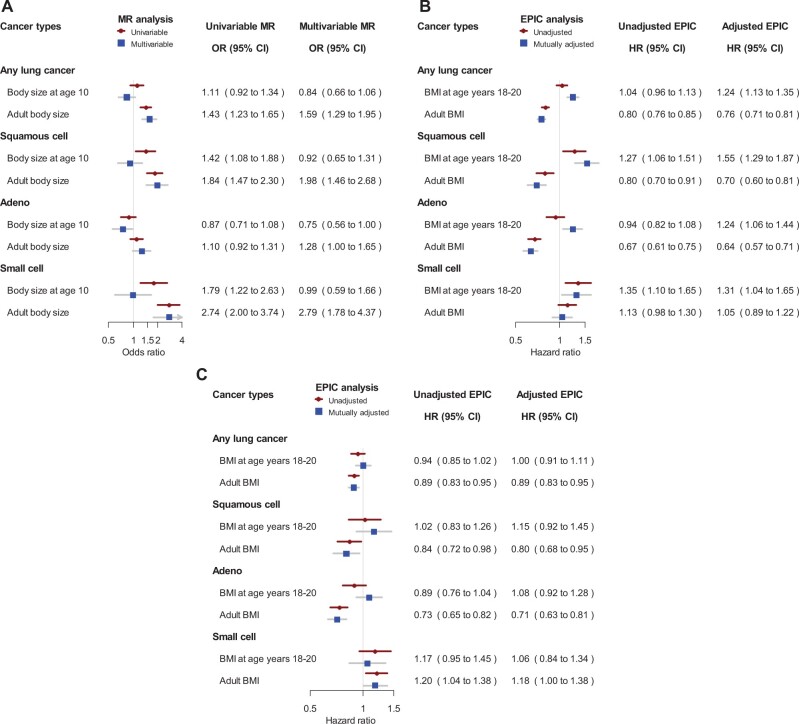
Mendelian randomization (MR) results and EPIC cohort results for lung cancer by histological subtypes. **A)** Odds ratios (ORs) and 95% confidence intervals (CIs) for category increase (ie, thinner than average, average, larger than average) in body size at age 10 years and adult body size before (univariable) and after (multivariable) mutual adjustment. **B)** Hazard ratios (HRs) for a 5-unit increase in BMI expressed in kg/m^2^ before (unadjusted) and after (adjusted) mutual adjustment. **C)** Hazard ratios for a 5-unit increase in BMI expressed in kg/m^2^ after adjustment for smoking before recruitment. BMI = body mass index; EPIC = European Prospective Investigation into Cancer and Nutrition.

Because studying correlated exposures may introduce the issues of pleiotropy and collinearity, we carried out a series of sensitivity analyses but found the observed risk associations robust (Supplementary Table 2 and Supplementary Figures 1-3, available online). The main cohort analysis deliberately did not account for other risk factors because most are likely to lie on the same causal pathway as obesity (Supplementary Table 3, available online), but we note that additional adjustments for smoking, alcohol, physical activity, and education at recruitment (Supplementary Table 4, available online) did not materially influence the associations estimates.

Owing to limitations in available data, we assessed body size at age 10 years for MR but BMI at age 18-20 years for the cohort analysis. BMI during childhood and early adulthood could have different importance in cancer etiology, which may explain some of the differences between MR and cohort results. Considering this caveat, we chose to limit our research question to whether the risk associations of early life and adult body size are independent and conservatively focused our interpretation on those cancers where consistent results are observed between the MR and cohort analyses.

In conclusion, early life BMI may be a risk factor for renal and endometrial cancer, but our findings indicate that the risk associations of early life BMI are not independent to that of adult BMI. This suggests that early life obesity contributes to risk of these 2 cancers through mechanistic pathways common to adult BMI.

## Funding

This work was supported by World Cancer Research Fund (WCRF UK) and Wereld Kanker Oderzoek Fonds (WKOF), as part of the World Cancer Research Fund International programme (IIG_2019_1995 to PB); and by Cancer Research UK [C18281/A29019]. The endometrial cancer genome-wide association analyses were supported by the National Health and Medical Research Council of Australia (APP552402, APP1031333, APP1109286, APP1111246, APP1061779); the US National Institutes of Health (R01-CA134958); European Research Council (EU FP7 Grant); Wellcome Trust Centre for Human Genetics (090532/Z/09Z); and Cancer Research UK. OncoArray genotyping of the Endometrial Cancer Association Consortium cases was performed with the generous assistance of the Ovarian Cancer Association Consortium (OCAC), which was funded through grants from the US National Institutes of Health (CA1X01HG007491-01 to CI Amos, U19-CA148112 to TA Sellers, R01-CA149429 to CM Phelan and R01-CA058598 to MT Goodman); Canadian Institutes of Health Research (MOP-86727 to LE Kelemen) and the Ovarian Cancer Research Fund (A Berchuck). We particularly thank the efforts of Cathy Phelan. OncoArray genotyping of the BCAC controls was funded by Genome Canada (GPH-129344); NIH (U19 CA148065); and Cancer UK (C1287/A16563). All studies and funders are listed in O’Mara et al. (2018). The authors thank the National Institute for Public Health and the Environment (RIVM), Bilthoven, the Netherlands, for their contribution and ongoing support to the EPIC study. The coordination of EPIC is financially supported by the European Commission (DG-SANCO) and the International Agency for Research on Cancer. The national cohorts are supported by Danish Cancer Society (Kræftens Bekæmpelse) (Denmark); Ligue Contre le Cancer; Institut Gustave Roussy; Mutuelle Générale de l’Education Nationale; Institut National de la Santé et de la Recherche Médicale (INSERM) (France); German Cancer Aid (Deutsche Krebshilfe); German Cancer Research Center (Deutsches Krebsforschungszentrum, DKFZ); Federal Ministry of Education and Research (Bundesministerium für Bildung und Forschung, BMBF) (Germany); Associazione Italiana per la Ricerca sul Cancro-AIRC-Italy and National Research Council (Italy); Dutch Ministry of Public Health, Welfare and Sports (VWS); Netherlands Cancer Registry (NKR); LK Research Funds; Dutch Prevention Funds; Dutch ZON (Zorg Onderzoek Nederland); World Cancer Research Fund (WCRF); Statistics Netherlands (the Netherlands); Health Research Fund (FISISCIII); Regional Governments of Andalucía, Asturias, Basque Country, Murcia, Navarra; the Catalan Institute of Oncology (Barcelona) (Spain); Swedish Cancer Society (Cancerfonden); Swedish Research Council (Vetenskapsrådet); County Councils of Skåne and Västerbotten (Sweden); Cancer Research UK (C570/A16491, C8221/A29017 and C8221/A19170 to EPIC-Oxford); Medical Research Council (MR/M012190/1 to EPIC-Oxford) (United Kingdom). Infrastructure support for the Department of Epidemiology and Biostatistics at Imperial College London (UK) was provided by the NIHR Imperial Biomedical Research Centre (BRC).

## Notes

**Role of the funders:** The funders had no role in the design of the study; the collection, analysis, and interpretation of the data; the writing of the manuscript; and the decision to submit the manuscript for publication.

**Disclosures:** TGR is employed part-time by Novo Nordisk outside of the research presented in this manuscript. The other authors declare no conflict of interests. SJC, who is a *JNCI* Associate Editor and co-author on this paper, was not involved in the editorial review or decision to publish the manuscript.

**Author contributions:** DM: Conceptualization, Methodology, Formal Analysis, Investigation, Writing Original Draft, Writing—Review & Editing. KSB, TGR: Conceptualization, Methodology, Formal Analysis, Investigation, Writing—Review & Editing. PF, MJG, NP, NM, SC, KKT, ER, DM: Conceptualization, Methodology, Investigation, Writing—Review & Editing. MPP, SJC, RJH, CIA, TAOM: Methodology, Formal Analysis, Investigation, Writing—Review & Editing. PA, FP, MRB, VK, AT, JH, APC, MDC, GS, CR, KBB, DA, AKH, HAW, MS, CB: Methodology, Investigation, Writing—Review & Editing. EW: Conceptualization, Methodology, Writing—Review & Editing. GDS: Conceptualization, Methodology, Investigation, Writing—Review & Editing. PB: Conceptualization, Methodology, Investigation, Supervision, Writing—Review & Editing. MJ: Conceptualization, Methodology, Investigation, Supervision, Writing Original Draft, Writing—Review & Editing.

**Acknowledgments:** The authors thank all the participants who took part in this research and the numerous institutions, researchers, clinicians, and technical and administrative staff who have made possible the many studies contributing to this work. In addition, the authors are grateful to Dr Joshua Atkins at the International Agency for Research on Cancer/World Health Organization for his contribution to the statistical analysis of this study.

**Disclaimers:** Where authors are identified as personnel of the International Agency for Research on Cancer/World Health Organization, the authors alone are responsible for the views expressed in this article, and they do not necessarily represent the decisions, policy, or views of the International Agency for Research on Cancer/World Health Organization.

## Supplementary Material

djac061_Supplementary_Data

## Data Availability

The data presented in this study are available on request from the corresponding author johanssonm@iarc.fr. This research was conducted accessing the UK Biobank data under application number 15825. Requests for the cancer data require formal approval by the principal investigators of each genetic consortium. For information on how to submit an application for gaining access to EPIC data and/or biospecimens, please follow the instructions at http://epic.iarc.fr/access/index.php.
