# Supplementary material for: Body Size at Different Ages and Risk of 6 Cancers: A Mendelian Randomization and Prospective Cohort Study
Source: J Natl Cancer Inst. 2022 Apr 19;114(9):1296–300. doi: 10.1093/jnci/djac061 (PMC9468294; doi:10.1093/jnci/djac061)

**Supplementary Material**

[Supplementary Methods 2](#_Toc92699948)

[References 6](#_Toc92699949)

[eTables 8](#_Toc92699950)

[eTable 1. Summary of genetic instruments for early life (age 10) and adult body size. 8](#_Toc92699951)

[eTable 2. Mendelian randomization results for different cancer sites and by histological subtypes. 9](#_Toc92699952)

[eTable 3. Cox regression results for different cancer sites and by histological subtypes. 15](#_Toc92699957)

[eTable 4. Cox regression results after adjustment for cancer risk factors at recruitment. 18](#_Toc92699962)

[eFigures 19](#_Toc92699966)

[eFigure 1. Leave-one-out multivariable Mendelian randomization using the inverse-variance weighted method for cancer of the colorectum (A), kidney (B), pancreas (C), lung (D), ovary (E), and endometrium (F). 19](#_Toc92699967)

[eFigure 2. Influential SNPs in univariable (UV) and multivariable (MV) Mendelian randomization for cancer of the colorectum (A), kidney (B), pancreas (C), lung (D), ovary (E), and endometrium (F). 20](#_Toc92699968)

[eFigure 3. Ridge regression estimates for body size and risk of cancer of the colorectum (A), kidney (B), pancreas (C), lung (D), ovary (E), and endometrium (F) and for body mass index in the EPIC cohort and risk of cancer of the colorectum (F), kidney (G), pancreas (H), lung (I), ovary (J), and endometrium (K). 21](#_Toc92699969)

# Supplementary Methods

*Genetic instruments for body size*

In this study we used genetic instruments for body size at different ages that we have previously identified using the data of UK Biobank and that we have recently validated [1]. Between 2006 and 2010 the UK Biobank study enrolled more than 500,000 individuals aged between 40 and 69 at baseline across 22 assessment centres in the United Kingdom. Data were collected based on clinical examinations, assays of biological samples, detailed information regarding self-reported health characteristics and genome-wide genotyping.

BMI was derived using height (measured in whole centimetres) and weight (measured to the nearest 0.1kg) at baseline. Participants were also asked ‘When you were 10 years old, compared to average would you describe yourself as thinner, plumper or about average?’. Only individuals with both body size measures were included in the analyses. To obtain risk estimates with similar interpretation for age 10 and adult age, adult BMI was converted into a categorical body size variable with 3 groups based on the same proportions as the early life body size variable (i.e. ‘thinner’ 33%, ‘about average’ 51% and ‘plumper’ 16%) which corresponded to cut-offs at BMI values 25 and 32. Effect estimates from our results can be interpreted as the increase in odds conferred per additive change in body size category.

As previously described [2], we used a Bayesian linear mixed model to evaluate the association between each genetic variant with each measure of body size in turn, while accounting for both relatedness and population stratification and adjusting for age at cohort entry, sex and type of genotyping array. Models for body size at age 10 were additionally adjusted for month of birth. Independent (r^2^<0.001 using genotype data from European individuals in the 1000 genomes project as a reference panel or more than 250 kb apart) genome-wide associated SNPs (P<5×10^−08^) were included in the genetic instruments for body size. Estimates were obtained for the overall sample and for women and men separately to obtain sex-specific instruments.

*Genetic predisposition to cancer*

We collected genome-wide association estimates for different obesity-related cancers from large-scale consortia. Recent genome-wide association studies (GWAS) based on many thousands of patients and controls of European ancestry are available for cancer of the colorectum [3], kidney [4], pancreas [5], lung [6], ovary [7], and endometrium [8, 9]. Genome-wide association estimates for subgroups with specific histology were available for lung, ovarian and endometrial cancer and sex-specific GWAS were available for kidney and pancreatic cancer. For endometrial cancer the overall GWAS - but not the histology-specific analysis - included data from the UK Biobank. We excluded SNPs with low imputation quality (imputation quality score <0.7, or for lung cancer imputation quality R^2^ < 0.3 or Info < 0.4 for each meta-analysis component) from all analyses.

*Direct measures of body size*

We investigated BMI (in kg/m^2^) at age 18-20 and during adulthood in relation to cancer risk in the EPIC cohort. EPIC is a prospective cohort of 521,324 study participants enrolled from 23 centres in 10 western European countries [10]. Detailed information on diet, lifestyle characteristics, anthropometric measurements, and medical history was collected at recruitment (1992—1999). We included the 239,860 participants from the following EPIC centres that collected self-reported weight at age 18-20 in the recruitment questionnaire: Varese (Italy), Naples (Italy), Cambridge (United Kingdom), Oxford (United Kingdom), Potsdam (Germany), Malmö (Sweden), Aarhus (Denmark), Copenhagen (Denmark) and Norway.

At cohort entry, weight was measured in most centres to the nearest 0.1 kg and corrected for clothing according to standardised procedures [11-13]. Exceptions were Oxford and Norway, where self-reported weight and height were collected and validated (for Norway) or corrected according to the results of the validation study (for Oxford) [14-16]. BMI at age 18-20 years was calculated from weight (in kg) at the age of 18-20 years and measured body height (in m) at cohort entry. BMI at cohort entry was calculated as weight at cohort entry divided by height squared (at cohort entry).

We excluded participants who were younger than 40 or older than 69 years of age at recruitment (n=35,515) to have the same range of age at recruitment as in UK Biobank. We further excluded participants with missing BMI at age 18-20 (n=17,899) and with extreme body size at age 18-20 or at recruitment (BMI<16 kg/m^2^, height<130 cm, waist circumference <40 or > 160 cm, and waist circumference <60 and BMI>25 kg/m^2^, n=1,085) to remove questionnaires with potential reporting errors.

Follow-up for incident cancers was based on population cancer registries in Denmark, Norway, Italy, Sweden, and the United Kingdom, and used a combination of methods (i.e. active follow-up by questionnaire and medical record linkages) in Germany. Mortality data were collected from regional or national registries.

*Statistical analysis*

We first performed two-sample MR analysis for each obesity-related cancer using the genetic instruments for body size (first sample) and the summary statistics for the cancer outcomes (second sample) [17]. Sex-specific instruments were used for ovarian and endometrial cancer and in analyses stratified by sex for other cancers. For all instruments we computed the *I*^2^ statistic to assess the NO Measurement Error (NOME) assumption [18]. Univariable MR analyses were conducted to investigate the associations of genetically predicted body size at age 10 and adult body size individually with each cancer. The main univariable MR estimates were obtained using the random-effects inverse-variance weighted (IVW) method. However, the estimated associations in univariable MR should be interpreted with caution because the instrument for childhood (or adult) body size include SNPs that are associated with adult (or childhood) body size too. If both exposures have direct effects on cancer risk the assumption of no horizontal pleiotropy is violated, resulting in potential bias in univariable estimates [19]. Multivariable MR analyses were undertaken to estimate the effect of early life body size that is not mediated by adult body size and a more accurate estimate of the effect of adult body size on each outcome [19]. Variants from the univariable analysis were used again here after undertaking further LD clumping (clumping cut-off r^2^=0.001 in the European individuals in the 1000 genomes project and clumping window of 250 kb) to account for correlation between instruments for childhood and adult BMI. The main multivariable MR effect estimates were obtained using the weighted regression-based method [20]. This method consists of regressing for all exposures (i.e. both early life and adult body size) the SNPs selected in the clumping step against the outcome together, weighting for the inverse variance of the outcome.

For the time-to-event analysis in the EPIC cohort, first diagnosis of cancer was identified for the EPIC participants. Patients with cancer history at cohort entry were excluded from the analysis and we identified cancer types, subtypes and morphologies according to the International Classification of Diseases for Oncology (ICD-O) for cancer of the colorectum (C18-C20), kidney (C64-C66, C68), pancreas (C25), lung (C33-C34; morphology code for squamous cell 8070, 8071, 8072, 8083; adeno 8140, 8144, 8230, 8250, 8253, 8254, 8256, 8257, 8260, 8265, 8333, 8480, 8551; small cell 8041, 8045), ovary (C56) and endometrium (C54; morphology code for endometrioid 8380) during follow-up. We estimated hazard ratios (HRs) for a 5-unit increase in BMI using Cox proportional hazards regression models, starting the follow-up at the date of the second weight measurement (cohort entry) and closing the follow-up on the date of first cancer diagnosis, death or centre-specific censoring (ranging between 2008, for Potsdam, and 2013, for Malmö). Participants diagnosed with cancers with ICD-O behaviour codes other than 3 (malignant, primary site) were not considered as cancer cases but their follow-up was censored at the cancer date except for participants with codes 0 (benign) and 1 (uncertain whether benign or malignant) which continued the follow-up. We could not perform histology-specific analysis for ovarian cancer because of the limited number of incident ovarian cancers. We used attained age as the time scale and all models were stratified by study centre. For the four cancers that present in both men and women the main models were stratified by sex; additionally, we ran sex-specific models and models with an interaction term between sex and BMI because the associations may vary between men and women. We estimated HRs for BMI at age 18-20 and adult BMI from models that included only one of these exposures and subsequently estimated HRs that were mutually adjusted for the other BMI exposure. In sensitivity analyses, we investigated other potential confounders of the association between BMI and cancer risk by adjusting for history of smoking before recruitment (ever smoking at age 18-20, lifetime number of cigarettes/day, smoking duration, current smoking, former smoking, time since quitting, interaction between former smoking and time since quitting), alcohol average lifetime intake, physical activity score, highest achieved education and age at menarche (for female cancers). We were particularly interested in adjusting for smoking history to evaluate the role of adult smoking as a confounder of the association between BMI at recruitment and cancer risk and a potential mediator of the effect of BMI at age 18-20 on cancer risk. Individuals with missing values in a covariate were excluded in the analysis adjusting for that covariate (i.e. complete case analysis).

For both the MR and cohort analysis we performed several additional statistical tests and sensitivity analyses. We assessed instrument strength by deriving F-statistics and conditional F-statistics for each MR analysis [21, 22]. To investigate the influence of potentially pleiotropic SNPs, we also performed the median method for univariable MR, the MR-Egger intercept test for testing the presence of unmeasured directional pleiotropy and performed a leave-one-out analysis [23-25]. Rucker’s Q' statistic and its individual components were used to test for global and individual pleiotropy after MR-Egger adjustment [26]. The list of traits that have been associated with the SNPs in our genetic instruments was obtained using PhenoScanner [27, 28]. To investigate influential values we computed Cook's Distance and we compared the number of associated traits for the most influential SNPs (Cook's Distance >4/number of SNPs in the model) after excluding traits that related to body fatness [29].

The correlation coefficient for the effects on body size at age 10 and adult body size for SNPs that were genome-wide significant for at least one of these traits was 0.74 and the correlation coefficient between BMI at age 18-20 and adult BMI in EPIC was 0.48. Therefore, collinearity could result in bias and inflated standard errors both in the multivariable MR and in the mutually adjusted Cox model. For each exposure we calculated the relative change in *χ*^2^ statistics for the coefficient after adjustment for the other BMI exposure to evaluate inflation of the standard errors. To further understand the impact of collinearity, we fitted ridge regression models. These models use L2 regularization that introduces a degree of bias to the regression estimates but also results in more accurate standard errors [30]. For these MR models we used inverse variance weights and did not introduce an intercept term so if the shrinkage parameter λ was null we obtained the multivariable IVW estimates. We used 10-fold cross-validation to estimate the best value of λ for each outcome and bootstrap to estimate 95% CIs.

We investigated the proportional hazards assumption of Cox regression models using the *χ*^2^ test based on Schoenfeld residuals. In a sensitivity analysis, we excluded the first two years of follow-up after date of recruitment to assess whether reverse causality had a major role in the observed associations between BMI at recruitment and cancer risk.

References

1. Richardson TG, Mykkanen J, Pahkala K*, et al.* Evaluating the direct effects of childhood adiposity on adult systemic metabolism: a multivariable Mendelian randomization analysis. Int J Epidemiol 2021;50(5):1580-1592.

2. Richardson TG, Sanderson E, Elsworth B*, et al.* Use of genetic variation to separate the effects of early and later life adiposity on disease risk: mendelian randomisation study. BMJ 2020;369:m1203.

3. Schmit SL, Edlund CK, Schumacher FR*, et al.* Novel Common Genetic Susceptibility Loci for Colorectal Cancer. J Natl Cancer Inst 2019;111(2):146-157.

4. Scelo G, Purdue MP, Brown KM*, et al.* Genome-wide association study identifies multiple risk loci for renal cell carcinoma. Nat Commun 2017;8:15724.

5. Klein AP, Wolpin BM, Risch HA*, et al.* Genome-wide meta-analysis identifies five new susceptibility loci for pancreatic cancer. Nat Commun 2018;9(1):556.

6. McKay JD, Hung RJ, Han Y*, et al.* Large-scale association analysis identifies new lung cancer susceptibility loci and heterogeneity in genetic susceptibility across histological subtypes. Nat Genet 2017;49(7):1126-1132.

7. Phelan CM, Kuchenbaecker KB, Tyrer JP*, et al.* Identification of 12 new susceptibility loci for different histotypes of epithelial ovarian cancer. Nat Genet 2017;49(5):680-691.

8. O’Mara TA, Glubb DM, Amant F*, et al.* Identification of nine new susceptibility loci for endometrial cancer. Nature Communications 2018;9(1):3166.

9. Kho PF, Mortlock S, Endometrial Cancer Association C*, et al.* Genetic analyses of gynecological disease identify genetic relationships between uterine fibroids and endometrial cancer, and a novel endometrial cancer genetic risk region at the WNT4 1p36.12 locus. Hum Genet 2021;140(9):1353-1365.

10. Riboli E, Hunt KJ, Slimani N*, et al.* European Prospective Investigation into Cancer and Nutrition (EPIC): study populations and data collection. Public Health Nutr 2002;5(6b):1113-1124.

11. Steins Bisschop CN, van Gils CH, Emaus MJ*, et al.* Weight change later in life and colon and rectal cancer risk in participants in the EPIC-PANACEA study. Am J Clin Nutr 2014;99(1):139-147.

12. Emaus MJ, van Gils CH, Bakker MF*, et al.* Weight change in middle adulthood and breast cancer risk in the EPIC-PANACEA study. Int J Cancer 2014;135(12):2887-2899.

13. Haftenberger M, Lahmann PH, Panico S*, et al.* Overweight, obesity and fat distribution in 50- to 64-year-old participants in the European Prospective Investigation into Cancer and Nutrition (EPIC). Public Health Nutr 2002;5(6b):1147-1162.

14. Lund E, Dumeaux V, Braaten T*, et al.* Cohort Profile: The Norwegian Women and Cancer Study—NOWAC—Kvinner og kreft. International Journal of Epidemiology 2007;37(1):36-41.

15. Spencer EA, Appleby PN, Davey GK*, et al.* Validity of self-reported height and weight in 4808 EPIC-Oxford participants. Public Health Nutr 2002;5(4):561-565.

16. Skeie G, Mode N, Henningsen M*, et al.* Validity of self-reported body mass index among middle-aged participants in the Norwegian Women and Cancer study. Clinical epidemiology 2015;7:313-323.

17. Pierce BL, Burgess S. Efficient design for Mendelian randomization studies: subsample and 2-sample instrumental variable estimators. Am J Epidemiol 2013;178(7):1177-1184.

18. Bowden J, Del Greco MF, Minelli C*, et al.* Assessing the suitability of summary data for two-sample Mendelian randomization analyses using MR-Egger regression: the role of the I2 statistic. Int J Epidemiol 2016;45(6):1961-1974.

19. Sanderson E, Davey Smith G, Windmeijer F*, et al.* An examination of multivariable Mendelian randomization in the single-sample and two-sample summary data settings. Int J Epidemiol 2019;48(3):713-727.

20. Burgess S, Dudbridge F, Thompson SG. Re: “Multivariable Mendelian Randomization: The Use of Pleiotropic Genetic Variants to Estimate Causal Effects”. American Journal of Epidemiology 2015;181(4):290-291.

21. Burgess S, Thompson SG, Collaboration CCG. Avoiding bias from weak instruments in Mendelian randomization studies. International Journal of Epidemiology 2011;40(3):755-764.

22. Sanderson E, Windmeijer F. A weak instrument F-test in linear IV models with multiple endogenous variables. Journal of Econometrics 2016;190(2):212-221.

23. Bowden J, Davey Smith G, Burgess S. Mendelian randomization with invalid instruments: effect estimation and bias detection through Egger regression. Int J Epidemiol 2015;44(2):512-525.

24. Bowden J, Davey Smith G, Haycock PC*, et al.* Consistent Estimation in Mendelian Randomization with Some Invalid Instruments Using a Weighted Median Estimator. Genet Epidemiol 2016;40(4):304-314.

25. Rees JMB, Wood AM, Burgess S. Extending the MR-Egger method for multivariable Mendelian randomization to correct for both measured and unmeasured pleiotropy. Stat Med 2017;36(29):4705-4718.

26. Bowden J, Hemani G, Davey Smith G. Invited Commentary: Detecting Individual and Global Horizontal Pleiotropy in Mendelian Randomization—A Job for the Humble Heterogeneity Statistic? American Journal of Epidemiology 2018;187(12):2681-2685.

27. Staley JR, Blackshaw J, Kamat MA*, et al.* PhenoScanner: a database of human genotype-phenotype associations. Bioinformatics 2016;32(20):3207-3209.

28. Kamat MA, Blackshaw JA, Young R*, et al.* PhenoScanner V2: an expanded tool for searching human genotype-phenotype associations. Bioinformatics 2019;35(22):4851-4853.

29. Cook RD. Detection of Influential Observation in Linear Regression. Technometrics 1977;19(1):15-18.

30. Hoerl AE, Kennard RW. Ridge Regression: Biased Estimation for Nonorthogonal Problems. Technometrics 1970;12(1):55-67.

# eTables

## eTable 1. Summary of genetic instruments for early life (age 10) and adult body size.

| Genetic instruments | No of SNPs | No of SNPs in LD | I^2^ statistic | % Variance explained |
| --- | --- | --- | --- | --- |
| Main instruments |  |  |  |  |
| Early life body size | 270 | 44 | 0.99 | 4.08 |
| Adult body size | 466 | 44 | 0.98 | 5.33 |
| Men-specific |  |  |  |  |
| Early life body size | 62 | 11 | 0.98 | 1.97 |
| Adult body size | 132 | 11 | 0.98 | 2.80 |
| Women-specific |  |  |  |  |
| Early life body size | 124 | 18 | 0.98 | 3.56 |
| Adult body size | 178 | 18 | 0.98 | 3.87 |

eTable 2. Mendelian randomization results for different cancer sites and by histological subtypes.

|  |  |  |  |  | **Univariable analysis** | | | | | **Multivariable analysis** | | | | | | | **Chi2 statistic** |
| --- | --- | --- | --- | --- | --- | --- | --- | --- | --- | --- | --- | --- | --- | --- | --- | --- | --- |
| **Outcome** | **method** | **Exposure** | **Cases** | **Controls** | **SNPs** | **F-statistic** | **OR** | **95%CI** | ***P*** | **method** | | **SNPs** | **conditional**  **F-statistics** | **OR** | **95%CI** | ***P*** | **%**  **change** |
| **Colorectal**  **Cancer** | IVW | Early life body size | 5,100 | 4,831 | 195 | 71.11 | 1.25 | 0.89-1.77 | 0.20 | IVW | | 452 | 10.99 | 0.76 | 0.46-1.26 | 0.29 | -31% |
|  | IVW | Adult body size | 5,100 | 4,831 | 343 | 56.96 | 1.77 | 1.30-2.41 | 3E-04 | IVW | 453 | | 13.94 | 2.03 | 1.29-3.20 | 2E-03 | -27% |
|  | MR-Egger | Early life body size | 5,100 | 4,831 | 195 |  | 1.67 | 0.81-3.42 | 0.17 | MR-Egger^a^ | | 452 |  | 0.84 | 0.48-1.48 | 0.55 |  |
|  | MR-Egger | Adult body size | 5,100 | 4,831 | 343 |  | 1.65 | 0.71-3.86 | 0.25 | MR-Egger^a^ | | 453 |  | 2.23 | 1.35-3.70 | 2E-03 |  |
|  | Median | Early life body size | 5,100 | 4,831 | 195 |  | 1.29 | 0.72-2.33 | 0.39 | MR-Egger^b^ | | 452 |  | 0.77 | 0.46-1.30 | 0.33 |  |
|  | Median | Adult body size | 5,100 | 4,831 | 343 |  | 1.49 | 0.87-2.55 | 0.14 | MR-Egger^b^ | | 453 |  | 2.38 | 1.17-4.80 | 0.02 |  |
| **Kidney**  **cancer** | IVW | Early life body size | 10,784 | 20,406 | 235 | 66.25 | 1.40 | 1.09-1.80 | 9E-03 | IVW | | 543 | 11.63 | 0.92 | 0.66-1.27 | 0.61 | -96% |
|  | IVW | Adult body size | 10,784 | 20,406 | 416 | 54.60 | 1.74 | 1.43-2.11 | 2E-08 | IVW | | 544 | 15.29 | 1.80 | 1.35-2.39 | 5E-05 | -48% |
|  | MR-Egger | Early life body size | 10,784 | 20,406 | 235 |  | 1.60† | 0.90-2.84 | 0.11 | MR-Egger^a^ | | 543 |  | 0.97 | 0.67-1.41 | 0.88 |  |
|  | MR-Egger | Adult body size | 10,784 | 20,406 | 416 |  | 2.67† | 1.50-4.73 | 9E-04 | MR-Egger^a^ | | 544 |  | 1.86 | 1.37-2.54 | 8E-05 |  |
|  | Median | Early life body size | 10,784 | 20,406 | 235 |  | 1.37 | 0.95-1.96 | 0.09 | MR-Egger^b^ | | 543 |  | 0.97 | 0.70-1.36 | 0.87 |  |
|  | Median | Adult body size | 10,784 | 20,406 | 416 |  | 2.14 | 1.52-3.01 | 1E-05 | MR-Egger^b^ | | 544 |  | 2.52 | 1.58-4.00 | 9E-05 |  |
| **Kidney**  **cancer**  ***in men*** | IVW | Early life body size | 3,227 | 4,915 | 54 | 64.80 | 1.58 | 0.86-2.93 | 0.14 | IVW | | 148 | 7.79 | 1.74 | 0.71-4.26 | 0.24 | -31% |
|  | IVW | Adult body size | 3,227 | 4,915 | 116 | 51.56 | 1.81 | 1.09-3.00 | 0.21 | IVW | | 148 | 10.31 | 1.22 | 0.56-2.69 | 0.62 | -95% |
|  | MR-Egger | Early life body size | 3,227 | 4,915 | 54 |  | 1.09 | 0.25-4.75 | 0.91 | MR-Egger^a^ | | 148 |  | 1.05* | 0.40-2.76 | 0.92 |  |
|  | MR-Egger | Adult body size | 3,227 | 4,915 | 116 |  | 1.11 | 0.23-5.28 | 0.90 | MR-Egger^a^ | | 148 |  | 0.78* | 0.33-1.82 | 0.56 |  |
|  | Median | Early life body size | 3,227 | 4,915 | 54 |  | 1.39 | 0.54-3.61 | 0.50 | MR-Egger^b^ | | 148 |  | 1.63 | 0.67-3.99 | 0.28 |  |
|  | Median | Adult body size | 3,227 | 4,915 | 116 |  | 1.33 | 0.57-3.11 | 0.50 | MR-Egger^b^ | | 148 |  | 0.56 | 0.15-2.12 | 0.40 |  |
| **Kidney**  **cancer**  ***in women*** | IVW | Early life body size | 1,992 | 3,095 | 115 | 68.13 | 1.31 | 0.78-2.21 | 0.31 | IVW | | 226 | 11.57 | 1.05 | 0.50-2.22 | 0.89 | -98% |
|  | IVW | Adult body size | 1,992 | 3,095 | 150 | 50.61 | 1.42 | 0.83-2.44 | 0.20 | IVW | | 226 | 12.19 | 1.29 | 0.63-2.65 | 0.48 | -70% |
|  | MR-Egger | Early life body size | 1,992 | 3,095 | 115 |  | 1.22 | 0.34-4.31 | 0.76 | MR-Egger^a^ | | 226 |  | 1.41 | 0.59-3.37 | 0.44 |  |
|  | MR-Egger | Adult body size | 1,992 | 3,095 | 150 |  | 2.06 | 0.39-10.94 | 0.40 | MR-Egger^a^ | | 226 |  | 1.57 | 0.72-3.42 | 0.25 |  |
|  | Median | Early life body size | 1,992 | 3,095 | 115 |  | 1.45 | 0.63-3.32 | 0.38 | MR-Egger^b^ | | 226 |  | 1.10 | 0.51-2.37 | 0.80 |  |
|  | Median | Adult body size | 1,992 | 3,095 | 150 |  | 1.76 | 0.76-4.11 | 0.19 | MR-Egger^b^ | | 226 |  | 1.67 | 0.54-5.18 | 0.38 |  |
| **Pancreatic**  **cancer** | IVW | Early life body size | 7,110 | 7,264 | 243 | 69.97 | 1.78 | 1.35-2.35 | 4E-05 | IVW | | 536 | 11.67 | 1.43 | 0.96-2.12 | 0.08 | -81% |
|  | IVW | Adult body size | 7,110 | 7,264 | 403 | 56.86 | 1.66 | 1.29-2.13 | 8E-05 | IVW | | 537 | 14.40 | 1.33 | 0.93-1.90 | 0.12 | -84% |
|  | MR-Egger | Early life body size | 7,110 | 7,264 | 243 |  | 1.71 | 0.94-3.08 | 0.08 | MR-Egger^a^ | | 536 |  | 1.55 | 0.99-2.44 | 0.06 |  |
|  | MR-Egger | Adult body size | 7,110 | 7,264 | 403 |  | 1.84† | 0.91-3.74 | 0.09 | MR-Egger^a^ | | 537 |  | 1.40 | 0.96-2.06 | 0.08 |  |
|  | Median | Early life body size | 7,110 | 7,264 | 243 |  | 2.07 | 1.35-3.16 | 8E-04 | MR-Egger^b^ | | 536 |  | 1.45 | 0.97-2.17 | 0.07 |  |
|  | Median | Adult body size | 7,110 | 7,264 | 403 |  | 2.29 | 1.57-3.34 | 2E-05 | MR-Egger^b^ | | 537 |  | 1.51 | 0.86-2.64 | 0.15 |  |
| **Pancreatic**  **cancer**  ***in men*** | IVW | Early life body size | 3,861 | 4,056 | 53 | 67.82 | 1.35 | 0.81-2.25 | 0.25 | IVW | | 144 | 7.74 | 1.47 | 0.66-3.28 | 0.35 | -33% |
|  | IVW | Adult body size | 3,861 | 4,056 | 114 | 52.15 | 1.22 | 0.76-1.95 | 0.40 | IVW | | 144 | 9.90 | 0.93 | 0.46-1.91 | 0.85 | -95% |
|  | MR-Egger | Early life body size | 3,861 | 4,056 | 53 |  | 1.23 | 0.38-3.98 | 0.73 | MR-Egger^a^ | | 144 |  | 1.30 | 0.54-3.13 | 0.56 |  |
|  | MR-Egger | Adult body size | 3,861 | 4,056 | 114 |  | 3.06 | 0.75-12.57 | 0.12 | MR-Egger^a^ | | 144 |  | 0.84 | 0.38-1.83 | 0.65 |  |
|  | Median | Early life body size | 3,861 | 4,056 | 53 |  | 1.45 | 0.66-3.20 | 0.35 | MR-Egger^b^ | | 144 |  | 1.51 | 0.67-3.38 | 0.32 |  |
|  | Median | Adult body size | 3,861 | 4,056 | 114 |  | 1.61 | 0.81-3.22 | 0.18 | MR-Egger^b^ | | 144 |  | 1.40 | 0.42-4.67 | 0.58 |  |
| **Pancreatic**  **cancer**  ***in women*** | IVW | Early life body size | 3,252 | 3,268 | 115 | 71.01 | 1.74 | 1.12-2.69 | 0.01 | IVW | | 224 | 11.34 | 1.50 | 0.79-2.86 | 0.22 | -75% |
|  | IVW | Adult body size | 3,252 | 3,268 | 147 | 51.98 | 1.59 | 0.98-2.57 | 0.06 | IVW | | 224 | 11.53 | 1.19 | 0.63-2.24 | 0.59 | -92% |
|  | MR-Egger | Early life body size | 3,252 | 3,268 | 115 |  | 5.65* | 2.16-14.80 | 6E-04 | MR-Egger^a^ | | 224 |  | 1.52 | 0.72-3.21 | 0.27 |  |
|  | MR-Egger | Adult body size | 3,252 | 3,268 | 147 |  | 4.73† | 1.21-18.55 | 0.03 | MR-Egger^a^ | | 224 |  | 1.20 | 0.60-2.39 | 0.60 |  |
|  | Median | Early life body size | 3,252 | 3,268 | 115 |  | 2.74 | 1.42-5.30 | 3E-03 | MR-Egger^b^ | | 224 |  | 1.62 | 0.84-3.12 | 0.15 |  |
|  | Median | Adult body size | 3,252 | 3,268 | 147 |  | 2.56 | 1.28-5.13 | 8E-03 | MR-Egger^b^ | | 224 |  | 1.92 | 0.73-5.02 | 0.18 |  |
| **Lung**  **cancer** | IVW | Early life body size | 29,266 | 56,450 | 238 | 65.33 | 1.11 | 0.92-1.34 | 0.26 | IVW | | 538 | 11.32 | 0.84 | 0.66-1.06 | 0.14 | 74% |
|  | IVW | Adult body size | 29,266 | 56,450 | 409 | 55.12 | 1.43 | 1.23-1.65 | 2E-06 | IVW | | 539 | 14.97 | 1.59 | 1.29-1.95 | 1E-05 | -14% |
|  | MR-Egger | Early life body size | 29,266 | 56,450 | 238 |  | 1.07† | 0.70-1.63 | 0.77 | MR-Egger^a^ | | 538 |  | 0.78 | 0.59-1.02 | 0.07 |  |
|  | MR-Egger | Adult body size | 29,266 | 56,450 | 409 |  | 1.48† | 0.96-2.28 | 0.08 | MR-Egger^a^ | | 539 |  | 1.52 | 1.21-1.90 | 3E-04 |  |
|  | Median | Early life body size | 29,266 | 56,450 | 238 |  | 0.96 | 0.76-1.22 | 0.75 | MR-Egger^b^ | | 538 |  | 0.84 | 0.65-1.07 | 0.15 |  |
|  | Median | Adult body size | 29,266 | 56,450 | 409 |  | 1.24 | 1.01-1.52 | 0.04 | MR-Egger^b^ | | 539 |  | 1.58 | 1.13-2.21 | **7E-03** |  |
| Squamous  cell | IVW | Early life body size | 7,426 | 55,627 | 236 | 64.03 | 1.42 | 1.08-1.88 | 0.01 | IVW | | 539 | 11.61 | 0.92 | 0.65-1.31 | 0.65 | -97% |
|  | IVW | Adult body size | 7,426 | 55,627 | 407 | 53.08 | 1.84 | 1.47-2.30 | 8E-08 | IVW | | 540 | 15.04 | 1.98 | 1.46-2.68 | 1E-05 | -32% |
|  | MR-Egger | Early life body size | 7,426 | 55,627 | 236 |  | 1.29† | 0.67-2.47 | 0.45 | MR-Egger^a^ | | 539 |  | 0.94 | 0.62-1.41 | 0.75 |  |
|  | MR-Egger | Adult body size | 7,426 | 55,627 | 407 |  | 2.69† | 1.30-5.56 | 0.01 | MR-Egger^a^ | | 540 |  | 2.00 | 1.43-2.79 | 4E-05 |  |
|  | Median | Early life body size | 7,426 | 55,627 | 236 |  | 1.30 | 0.87-1.92 | 0.20 | MR-Egger^b^ | | 539 |  | 0.94 | 0.65-1.34 | 0.72 |  |
|  | Median | Adult body size | 7,426 | 55,627 | 407 |  | 1.60 | 1.17-2.18 | 3E-03 | MR-Egger^b^ | | 540 |  | 2.11 | 1.26-3.54 | 4E-03 |  |
| Adeno | IVW | Early life body size | 11,273 | 55,483 | 236 | 68.23 | 0.87 | 0.71-1.08 | 0.22 | IVW | | 544 | 11.42 | 0.75 | 0.56-1.00 | 0.05 | 163% |
|  | IVW | Adult body size | 11,273 | 55,483 | 413 | 55.64 | 1.10 | 0.92-1.31 | 0.28 | IVW | | 545 | 14.73 | 1.28 | 1.00-1.65 | 0.05 | 230% |
|  | MR-Egger | Early life body size | 11,273 | 55,483 | 236 |  | 0.97† | 0.60-1.54 | 0.88 | MR-Egger^a^ | | 544 |  | 0.73 | 0.52-1.01 | 0.06 |  |
|  | MR-Egger | Adult body size | 11,273 | 55,483 | 413 |  | 1.07† | 0.64-1.80 | 0.78 | MR-Egger^a^ | | 545 |  | 1.26 | 0.96-1.66 | 0.10 |  |
|  | Median | Early life body size | 11,273 | 55,483 | 236 |  | 0.87 | 0.64-1.20 | 0.39 | MR-Egger^b^ | | 544 |  | 0.76 | 0.57-1.02 | 0.06 |  |
|  | Median | Adult body size | 11,273 | 55,483 | 413 |  | 1.00 | 0.77-1.28 | 0.98 | MR-Egger^b^ | | 545 |  | 1.42 | 0.95-2.12 | 0.09 |  |
| Small cell | IVW | Early life body size | 2,664 | 21,444 | 235 | 67.46 | 1.79 | 1.22-2.63 | 3E-03 | IVW | | 543 | 11.43 | 0.99 | 0.59-1.66 | 0.98 | -100% |
|  | IVW | Adult body size | 2,664 | 21,444 | 413 | 55.03 | 2.74 | 2.00-3.74 | 2E-10 | IVW | | 544 | 14.84 | 2.79 | 1.78-4.37 | 8E-06 | -50% |
|  | MR-Egger | Early life body size | 2,664 | 21,444 | 235 |  | 2.19† | 0.94-5.09 | 0.07 | MR-Egger^a^ | | 543 |  | 0.88 | 0.49-1.59 | 0.68 |  |
|  | MR-Egger | Adult body size | 2,664 | 21,444 | 413 |  | 2.61† | 1.06-6.48 | 0.04 | MR-Egger^a^ | | 544 |  | 2.59 | 1.59-4.21 | 1E-04 |  |
|  | Median | Early life body size | 2,664 | 21,444 | 235 |  | 1.24 | 0.68-2.27 | 0.49 | MR-Egger^b^ | | 543 |  | 0.99 | 0.59-1.67 | 0.97 |  |
|  | Median | Adult body size | 2,664 | 21,444 | 413 |  | 2.39 | 1.44-3.96 | 7E-04 | MR-Egger^b^ | | 544 |  | 2.73 | 1.32-5.66 | 7E-03 |  |
| **Ovarian**  **cancer** | IVW | Early life body size | 25,509 | 40,941 | 110 | 64.74 | 1.18 | 0.96-1.44 | 0.12 | IVW | | 222 | 11.13 | 1.09 | 0.83-1.42 | 0.54 | -85% |
|  | IVW | Adult body size | 25,509 | 40,941 | 150 | 49.65 | 1.20 | 1.00-1.44 | 0.05 | IVW | | 223 | 12.65 | 1.13 | 0.88-1.45 | 0.33 | -76% |
|  | MR-Egger | Early life body size | 25,509 | 40,941 | 110 |  | 2.30*† | 1.41-3.75 | 1E-03 | MR-Egger^a^ | | 222 |  | 1.22 | 0.88-1.67 | 0.23 |  |
|  | MR-Egger | Adult body size | 25,509 | 40,941 | 150 |  | 2.23*† | 1.29-3.85 | 5E-03 | MR-Egger^a^ | | 223 |  | 1.21 | 0.92-1.59 | 0.16 |  |
|  | Median | Early life body size | 25,509 | 40,941 | 110 |  | 1.47 | 1.13-1.92 | 4E-03 | MR-Egger^b^ | | 222 |  | 1.19* | 0.91-1.57 | 0.20 |  |
|  | Median | Adult body size | 25,509 | 40,941 | 150 |  | 1.24 | 0.96-1.59 | 0.09 | MR-Egger^b^ | | 223 |  | 1.81* | 1.21-2.70 | 4E-03 |  |
| Serous | IVW | Early life body size | 16,003 | 40,941 | 110 | 64.74 | 1.10 | 0.87-1.39 | 0.43 | IVW | | 222 | 11.13 | 1.06 | 0.77-1.46 | 0.73 | -81% |
|  | IVW | Adult body size | 16,003 | 40,941 | 150 | 49.65 | 1.15 | 0.92-1.43 | 0.21 | IVW | | 223 | 12.65 | 1.10 | 0.81-1.48 | 0.54 | -76% |
|  | MR-Egger | Early life body size | 16,003 | 40,941 | 110 |  | 1.96*† | 1.09-3.50 | 0.03 | MR-Egger^a^ | | 222 |  | 1.17 | 0.80-1.72 | 0.41 |  |
|  | MR-Egger | Adult body size | 16,003 | 40,941 | 150 |  | 1.76† | 0.91-3.43 | 0.10 | MR-Egger^a^ | | 223 |  | 1.17 | 0.85-1.62 | 0.34 |  |
|  | Median | Early life body size | 16,003 | 40,941 | 110 |  | 1.21 | 0.87-1.68 | 0.25 | MR-Egger^b^ | | 222 |  | 1.15* | 0.83-1.59 | 0.41 |  |
|  | Median | Adult body size | 16,003 | 40,941 | 150 |  | 1.25 | 0.91-1.71 | 0.16 | MR-Egger^b^ | | 223 |  | 1.66* | 1.02-2.68 | 0.04 |  |
| Serous  high grade | IVW | Early life body size | 13,037 | 40,941 | 110 | 64.74 | 1.11 | 0.87-1.41 | 0.40 | IVW | | 222 | 11.13 | 1.05 | 0.75-1.46 | 0.78 | -89% |
|  | IVW | Adult body size | 13,037 | 40,941 | 150 | 49.65 | 1.19 | 0.95-1.49 | 0.13 | IVW | | 223 | 12.65 | 1.14 | 0.84-1.54 | 0.42 | -71% |
|  | MR-Egger | Early life body size | 13,037 | 40,941 | 110 |  | 1.91† | 1.06-3.46 | 0.03 | MR-Egger^a^ | | 222 |  | 1.12 | 0.76-1.66 | 0.56 |  |
|  | MR-Egger | Adult body size | 13,037 | 40,941 | 150 |  | 1.75† | 0.88-3.49 | 0.11 | MR-Egger^a^ | | 223 |  | 1.19 | 0.85-1.66 | 0.31 |  |
|  | Median | Early life body size | 13,037 | 40,941 | 110 |  | 1.28 | 0.90-1.81 | 0.17 | MR-Egger^b^ | | 222 |  | 1.13 | 0.81-1.58 | 0.48 |  |
|  | Median | Adult body size | 13,037 | 40,941 | 150 |  | 1.29 | 0.93-1.78 | 0.12 | MR-Egger^b^ | | 223 |  | 1.65 | 1.01-2.71 | 0.05 |  |
| Serous  low grade | IVW | Early life body size | 1,012 | 40,941 | 110 | 64.74 | 1.03 | 0.52-2.04 | 0.93 | IVW | | 222 | 11.13 | 1.26 | 0.54-2.95 | 0.60 | 3217% |
|  | IVW | Adult body size | 1,012 | 40,941 | 150 | 49.65 | 0.74 | 0.42-1.30 | 0.29 | IVW | | 223 | 12.65 | 0.69 | 0.31-1.53 | 0.36 | -23% |
|  | MR-Egger | Early life body size | 1,012 | 40,941 | 110 |  | 3.07† | 0.56-16.72 | 0.20 | MR-Egger^a^ | | 222 |  | 2.14 | 0.78-5.86 | 0.14 |  |
|  | MR-Egger | Adult body size | 1,012 | 40,941 | 150 |  | 2.15 | 0.38-12.14 | 0.39 | MR-Egger^a^ | | 223 |  | 0.97 | 0.41-2.28 | 0.93 |  |
|  | Median | Early life body size | 1,012 | 40,941 | 110 |  | 1.11 | 0.36-3.46 | 0.85 | MR-Egger^b^ | | 222 |  | 1.50 | 0.62-3.58 | 0.37 |  |
|  | Median | Adult body size | 1,012 | 40,941 | 150 |  | 0.81 | 0.30-2.16 | 0.67 | MR-Egger^b^ | | 223 |  | 1.66 | 0.46-6.03 | 0.44 |  |
| Serous low  malignant potential | IVW | Early life body size | 1,954 | 40,941 | 110 | 64.74 | 1.88 | 1.18-3.01 | 8E-03 | IVW | | 222 | 11.13 | 1.43 | 0.77-2.65 | 0.25 | -82% |
|  | IVW | Adult body size | 1,954 | 40,941 | 150 | 49.65 | 2.12 | 1.39-3.23 | 5E-04 | IVW | | 223 | 12.65 | 1.72 | 0.97-3.05 | 0.06 | -72% |
|  | MR-Egger | Early life body size | 1,954 | 40,941 | 110 |  | 6.46* | 2.04-20.41 | 2E-03 | MR-Egger^a^ | | 222 |  | 1.97 | 0.95-4.08 | 0.07 |  |
|  | MR-Egger | Adult body size | 1,954 | 40,941 | 150 |  | 4.36 | 1.19-16.05 | 0.03 | MR-Egger^a^ | | 223 |  | 2.10 | 1.12-3.92 | 0.02 |  |
|  | Median | Early life body size | 1,954 | 40,941 | 110 |  | 3.02 | 1.42-6.43 | 4E-03 | MR-Egger^b^ | | 222 |  | 1.64 | 0.87-3.09 | 0.12 |  |
|  | Median | Adult body size | 1,954 | 40,941 | 150 |  | 2.44 | 1.23-4.82 | 0.01 | MR-Egger^b^ | | 223 |  | 3.41 | 1.34-8.63 | 0.01 |  |
| Mucinous | IVW | Early life body size | 2,566 | 40,941 | 110 | 64.74 | 1.45 | 0.96-2.17 | 0.08 | IVW | | 222 | 11.13 | 1.41 | 0.83-2.41 | 0.21 | -49% |
|  | IVW | Adult body size | 2,566 | 40,941 | 150 | 49.65 | 1.26 | 0.87-1.82 | 0.22 | IVW | | 223 | 12.65 | 0.98 | 0.59-1.60 | 0.92 | -99% |
|  | MR-Egger | Early life body size | 2,566 | 40,941 | 110 |  | 2.26 | 0.81-6.28 | 0.12 | MR-Egger^a^ | | 222 |  | 1.76 | 0.94-3.32 | 0.08 |  |
|  | MR-Egger | Adult body size | 2,566 | 40,941 | 150 |  | 3.00 | 0.97-9.29 | 0.06 | MR-Egger^a^ | | 223 |  | 1.12 | 0.65-1.93 | 0.67 |  |
|  | Median | Early life body size | 2,566 | 40,941 | 110 |  | 1.53 | 0.79-2.99 | 0.21 | MR-Egger^b^ | | 222 |  | 1.55 | 0.90-2.68 | 0.12 |  |
|  | Median | Adult body size | 2,566 | 40,941 | 150 |  | 1.61 | 0.88-2.93 | 0.12 | MR-Egger^b^ | | 223 |  | 1.57 | 0.70-3.52 | 0.27 |  |
| Mucinous  invasive | IVW | Early life body size | 1,417 | 40,941 | 110 | 64.74 | 1.30 | 0.78-2.19 | 0.31 | IVW | | 222 | 11.13 | 1.24 | 0.61-2.54 | 0.55 | -65% |
|  | IVW | Adult body size | 1,417 | 40,941 | 150 | 49.65 | 1.20 | 0.73-1.97 | 0.46 | IVW | | 223 | 12.65 | 0.97 | 0.50-1.88 | 0.92 | -98% |
|  | MR-Egger | Early life body size | 1,417 | 40,941 | 110 |  | 4.86* | 1.36-17.32 | 0.02 | MR-Egger^a^ | | 222 |  | 1.84 | 0.79-4.28 | 0.16 |  |
|  | MR-Egger | Adult body size | 1,417 | 40,941 | 150 |  | 7.49* | 1.68-33.37 | 9E-03 | MR-Egger^a^ | | 223 |  | 1.24 | 0.60-2.55 | 0.56 |  |
|  | Median | Early life body size | 1,417 | 40,941 | 110 |  | 1.56 | 0.71-3.44 | 0.27 | MR-Egger^b^ | | 222 |  | 1.54* | 0.74-3.18 | 0.24 |  |
|  | Median | Adult body size | 1,417 | 40,941 | 150 |  | 1.49 | 0.70-3.21 | 0.30 | MR-Egger^b^ | | 223 |  | 2.84* | 0.97-8.26 | 0.06 |  |
| Mucinous low  malignant potential | IVW | Early life body size | 1,149 | 40,941 | 110 | 64.74 | 1.50 | 0.82-2.75 | 0.19 | IVW | | 222 | 11.13 | 1.49 | 0.72-3.10 | 0.28 | -32% |
|  | IVW | Adult body size | 1,149 | 40,941 | 150 | 49.65 | 1.29 | 0.77-2.16 | 0.34 | IVW | | 223 | 12.65 | 0.99 | 0.50-1.95 | 0.97 | -100% |
|  | MR-Egger | Early life body size | 1,149 | 40,941 | 110 |  | 0.94 | 0.21-4.28 | 0.93 | MR-Egger^a^ | | 222 |  | 1.57 | 0.66-3.73 | 0.31 |  |
|  | MR-Egger | Adult body size | 1,149 | 40,941 | 150 |  | 1.08 | 0.22-5.33 | 0.92 | MR-Egger^a^ | | 223 |  | 1.02 | 0.48-2.14 | 0.96 |  |
|  | Median | Early life body size | 1,149 | 40,941 | 110 |  | 0.95 | 0.37-2.42 | 0.91 | MR-Egger^b^ | | 222 |  | 1.48 | 0.70-3.13 | 0.31 |  |
|  | Median | Adult body size | 1,149 | 40,941 | 150 |  | 0.88 | 0.37-2.09 | 0.78 | MR-Egger^b^ | | 223 |  | 0.93 | 0.31-2.82 | 0.90 |  |
| Low malignant  potential | IVW | Early life body size | 6,206 | 40,941 | 110 | 64.74 | 1.74 | 1.22-2.50 | 2E-03 | IVW | | 222 | 11.13 | 1.44 | 0.89-2.33 | 0.14 | -76% |
|  | IVW | Adult body size | 6,206 | 40,941 | 150 | 49.65 | 1.78 | 1.27-2.51 | 9E-04 | IVW | | 223 | 12.65 | 1.43 | 0.92-2.24 | 0.12 | -78% |
|  | MR-Egger | Early life body size | 6,206 | 40,941 | 110 |  | 3.11 | 1.27-7.62 | 0.01 | MR-Egger^a^ | | 222 |  | 1.79 | 1.01-3.16 | 0.04 |  |
|  | MR-Egger | Adult body size | 6,206 | 40,941 | 150 |  | 2.49 | 0.87-7.13 | 0.09 | MR-Egger^a^ | | 223 |  | 1.64 | 1.01-2.68 | 0.04 |  |
|  | Median | Early life body size | 6,206 | 40,941 | 110 |  | 2.08 | 1.13-3.82 | 0.02 | MR-Egger^b^ | | 222 |  | 1.55 | 0.94-2.53 | 0.08 |  |
|  | Median | Adult body size | 6,206 | 40,941 | 150 |  | 1.93 | 1.09-3.42 | 0.02 | MR-Egger^b^ | | 223 |  | 2.05 | 0.99-4.25 | 0.05 |  |
| Clear cell | IVW | Early life body size | 1,366 | 40,941 | 110 | 64.74 | 1.26 | 0.75-2.13 | 0.39 | IVW | | 222 | 11.13 | 1.22 | 0.61-2.44 | 0.57 | -58% |
|  | IVW | Adult body size | 1,366 | 40,941 | 150 | 49.65 | 1.28 | 0.78-2.08 | 0.33 | IVW | | 223 | 12.65 | 1.03 | 0.54-1.97 | 0.92 | -99% |
|  | MR-Egger | Early life body size | 1,366 | 40,941 | 110 |  | 6.55* | 1.83-23.44 | 5E-03 | MR-Egger^a^ | | 222 |  | 1.80 | 0.79-4.08 | 0.16 |  |
|  | MR-Egger | Adult body size | 1,366 | 40,941 | 150 |  | 3.02 | 0.67-13.47 | 0.15 | MR-Egger^a^ | | 223 |  | 1.32 | 0.66-2.66 | 0.44 |  |
|  | Median | Early life body size | 1,366 | 40,941 | 110 |  | 1.78 | 0.81-3.91 | 0.15 | MR-Egger^b^ | | 222 |  | 1.39 | 0.68-2.83 | 0.36 |  |
|  | Median | Adult body size | 1,366 | 40,941 | 150 |  | 1.55 | 0.72-3.33 | 0.27 | MR-Egger^b^ | | 223 |  | 1.99 | 0.70-5.67 | 0.20 |  |
| Endometrioid | IVW | Early life body size | 2,810 | 40,941 | 110 | 64.74 | 1.57 | 1.07-2.31 | 0.02 | IVW | | 222 | 11.13 | 1.11 | 0.67-1.85 | 0.68 | -97% |
|  | IVW | Adult body size | 2,810 | 40,941 | 150 | 49.65 | 1.57 | 1.08-2.27 | 0.02 | IVW | | 223 | 12.65 | 1.53 | 0.96-2.46 | 0.08 | -45% |
|  | MR-Egger | Early life body size | 2,810 | 40,941 | 110 |  | 2.18 | 0.83-5.74 | 0.12 | MR-Egger^a^ | | 222 |  | 1.20 | 0.65-2.19 | 0.56 |  |
|  | MR-Egger | Adult body size | 2,810 | 40,941 | 150 |  | 6.47* | 2.12-19.70 | 1E-03 | MR-Egger^a^ | | 223 |  | 1.61 | 0.96-2.69 | 0.07 |  |
|  | Median | Early life body size | 2,810 | 40,941 | 110 |  | 1.50 | 0.80-2.80 | 0.20 | MR-Egger^b^ | | 222 |  | 1.26* | 0.75-2.12 | 0.38 |  |
|  | Median | Adult body size | 2,810 | 40,941 | 150 |  | 1.53 | 0.84-2.79 | 0.17 | MR-Egger^b^ | | 223 |  | 2.89* | 1.35-6.21 | 6E-03 |  |
| **Endometrial** | IVW | Early life body size | 12,906 | 108,979 | 121 | 69.71 | 1.61 | 1.23-2.11 | 5E-04 | IVW | | 241 | 11.36 | 0.96 | 0.70-1.32 | 0.81 | -100% |
|  | IVW | Adult body size | 12,906 | 108,979 | 162 | 51.10 | 2.19 | 1.79-2.69 | 5E-14 | IVW | | 241 | 12.08 | 2.16 | 1.59-2.93 | 8E-07 | -57% |
|  | MR-Egger | Early life body size | 12,906 | 108,979 | 121 |  | 1.81† | 0.98-3.37 | 0.06 | MR-Egger^a^ | | 241 |  | 1.08 | 0.75-1.55 | 0.68 |  |
|  | MR-Egger | Adult body size | 12,906 | 108,979 | 162 |  | 3.68† | 2.02-6.71 | 3E-05 | MR-Egger^a^ | | 241 |  | 2.36 | 1.69-3.30 | 5E-07 |  |
|  | Median | Early life body size | 12,906 | 108,979 | 121 |  | 1.97 | 1.47-2.64 | 6E-06 | MR-Egger^b^ | | 241 |  | 0.98 | 0.71-1.36 | 0.92 |  |
|  | Median | Adult body size | 12,906 | 108,979 | 162 |  | 2.24 | 1.66-3.02 | 1E-07 | MR-Egger^b^ | | 241 |  | 2.45 | 1.52-3.96 | 2E-04 |  |
| Endometrioid | IVW | Early life body size | 8,758 | 46,126 | 121 | 69.71 | 1.66 | 1.23-2.24 | 9E-04 | IVW | | 241 | 11.36 | 0.95 | 0.66-1.37 | 0.79 | -99% |
|  | IVW | Adult body size | 8,758 | 46,126 | 162 | 51.10 | 2.34 | 1.85-2.96 | 2E-12 | IVW | | 241 | 12.08 | 2.30 | 1.62-3.28 | 3E-06 | -57% |
|  | MR-Egger | Early life body size | 8,758 | 46,126 | 121 |  | 1.74† | 0.87-3.46 | 0.12 | MR-Egger^a^ | | 241 |  | 1.06 | 0.69-1.61 | 0.80 |  |
|  | MR-Egger | Adult body size | 8,758 | 46,126 | 162 |  | 3.20† | 1.59-6.43 | 1E-03 | MR-Egger^a^ | | 241 |  | 2.50 | 1.70-3.67 | 3E-06 |  |
|  | Median | Early life body size | 8,758 | 46,126 | 121 |  | 2.21 | 1.54-3.18 | 2E-05 | MR-Egger^b^ | | 241 |  | 0.95 | 0.66-1.39 | 0.81 |  |
|  | Median | Adult body size | 8,758 | 46,126 | 162 |  | 2.27 | 1.62-3.16 | 1E-06 | MR-Egger^b^ | | 241 |  | 2.34 | 1.35-4.07 | 2E-03 |  |
| Non-Endometrioid | IVW | Early life body size | 1,230 | 35,447 | 120 | 69.96 | 1.55 | 0.89-2.67 | 0.12 | IVW | | 240 | 11.41 | 1.71 | 0.82-3.57 | 0.15 | -16% |
|  | IVW | Adult body size | 1,230 | 35,447 | 162 | 51.10 | 1.14 | 0.69-1.87 | 0.60 | IVW | | 240 | 12.18 | 0.86 | 0.42-1.76 | 0.69 | -40% |
|  | MR-Egger | Early life body size | 1,230 | 35,447 | 120 |  | 1.22 | 0.34-4.31 | 0.76 | MR-Egger^a^ | | 240 |  | 2.20 | 0.95-5.13 | 0.07 |  |
|  | MR-Egger | Adult body size | 1,230 | 35,447 | 162 |  | 10.17* | 2.38-43.45 | 2E-03 | MR-Egger^a^ | | 240 |  | 1.05 | 0.48-2.29 | 0.90 |  |
|  | Median | Early life body size | 1,230 | 35,447 | 120 |  | 1.19 | 0.53-2.69 | 0.67 | MR-Egger^b^ | | 240 |  | 1.76 | 0.83-3.74 | 0.14 |  |
|  | Median | Adult body size | 1,230 | 35,447 | 162 |  | 1.30 | 0.57-2.94 | 0.53 | MR-Egger^b^ | | 240 |  | 1.04 | 0.34-3.17 | 0.94 |  |

* P-value for Egger intercept <0.05

† P-value test for global pleiotropy based on Rucker's Q’ <0.05

^a^ Early life body size is the reference exposure

^b^ Adult body size is the reference exposure

eTable 3. Cox regression results for different cancer sites and by histological subtypes.

|  |  |  | **Main analysis** | | | | | | | |  | **Sensitivity analysis - excluding the 1^st^ two years after recruitment** | | | | | | | | |
| --- | --- | --- | --- | --- | --- | --- | --- | --- | --- | --- | --- | --- | --- | --- | --- | --- | --- | --- | --- | --- |
|  |  |  |  |  | **Exposure-specific**  **models^a^** | | | **Mutually-adjusted**  **model^b^** | | |  |  |  | **Exposure-specific**  **models^a^** | | | **Mutually-adjusted**  **model^b^** | | | |
| **Outcome** | **Sample^c^** | **Exposure^d^** | **N** | **N cancer** | **HR** | **95%CI** | ***P*** | **HR** | **95%CI** | ***P*** |  | **N** | **N cancer** | **HR** | **95%CI** | ***P*** | **HR** | **95%CI** | ***P*** | |
| **Colorectal**  **cancer** | All | BMI age 18-20 | 185,361 | 3,104 | 1.06 | 0.99-1.13 | 0.12 | 0.97 | 0.90-1.04 | 0.40 |  | 182,315 | 2,877 | 1.05 | 0.98-1.13 | 0.16 | 0.97 | 0.89-1.05 | 0.43 | |
|  | All | Adult BMI | 185,361 | 3,104 | 1.13 | 1.08-1.18 | 7E-08 | 1.14 | 1.08-1.19 | 2E-07 |  | 182,315 | 2,877 | 1.12 | 1.07-1.17 | 9E-07 | 1.13 | 1.07-1.19 | 2E-06 | |
|  | All | BMI age 18-20*Sex | 185,361 | 3,104 | 1.00 | 0.87-1.15 | 0.99 | 0.93 | 0.80-1.08 | 0.35 |  | 182,315 | 2,877 | 1.00 | 0.86-1.15 | 0.96 | 0.92 | 0.78-1.08 | 0.31 | |
|  | All | Adult BMI * Sex | 185,361 | 3,104 | 1.11 | 1.01-1.21 | 0.02 | 1.14 | 1.03-1.26 | 0.01 |  | 182,315 | 2,877 | 1.11 | 1.01-1.22 | 0.02 | 1.14 | 1.03-1.27 | 0.01 | |
|  | Men | BMI age 18-20 | 59,200 | 1,391 | 1.05 | 0.94-1.17 | 0.36 | 0.92 | 0.82-1.04 | 0.18 |  | 58,007 | 1,288 | 1.05 | 0.94-1.17 | 0.43 | 0.92 | 0.81-1.04 | 0.19 | |
|  | Men | Adult BMI | 59,200 | 1,391 | 1.20 | 1.11-1.29 | 1E-06 | 1.23 | 1.13-1.33 | 6E-07 |  | 58,007 | 1,288 | 1.19 | 1.11-1.29 | 4E-06 | 1.22 | 1.13-1.33 | 2E-06 | |
|  | Women | BMI age 18-20 | 126,161 | 1,713 | 1.06 | 0.97-1.16 | 0.20 | 1.00 | 0.90-1.10 | 0.95 |  | 124,308 | 1,589 | 1.06 | 0.96-1.16 | 0.25 | 1.00 | 0.90-1.11 | 0.99 | |
|  | Women | Adult BMI | 126,161 | 1,713 | 1.09 | 1.03-1.15 | 2E-03 | 1.09 | 1.03-1.16 | 5E-03 |  | 124,308 | 1,589 | 1.08 | 1.02-1.14 | 7E-03 | 1.08 | 1.02-1.15 | 0.01 | |
| **Kidney**  **cancer** | All | BMI age 18-20 | 185,361 | 579 | 1.16 | 1.00-1.36 | 0.05 | 0.98 | 0.82-1.16 | 0.80 |  | 182,315 | 513 | 1.21 | 1.03-1.42 | 0.02 | 1.01 | 0.84-1.21 | 0.94 | |
|  | All | Adult BMI | 185,361 | 579 | 1.28 | 1.16-1.40 | 6E-07 | 1.28 | 1.15-1.43 | 5E-06 |  | 182,315 | 513 | 1.30 | 1.18-1.44 | 3E-07 | 1.30 | 1.16-1.46 | 6E-06 | |
|  | All | BMI age 18-20*Sex | 185,361 | 579 | 0.75 | 0.56-1.01 | 0.06 | 0.70 | 0.50-0.98 | 0.04 |  | 182,315 | 513 | 0.81 | 0.59-1.11 | 0.20 | 0.75 | 0.52-1.07 | 0.11 | |
|  | All | Adult BMI * Sex | 185,361 | 579 | 1.03 | 0.85-1.25 | 0.77 | 1.14 | 0.92-1.42 | 0.22 |  | 182,315 | 513 | 1.07 | 0.87-1.31 | 0.54 | 1.16 | 0.92-1.46 | 0.20 | |
|  | Men | BMI age 18-20 | 59,200 | 310 | 0.98 | 0.78-1.23 | 0.84 | 0.79 | 0.61-1.01 | 0.06 |  | 58,007 | 275 | 1.06 | 0.83-1.34 | 0.66 | 0.84 | 0.65-1.09 | 0.20 | |
|  | Men | Adult BMI | 59,200 | 310 | 1.29 | 1.12-1.50 | 6E-04 | 1.39 | 1.18-1.64 | 9E-05 |  | 58,007 | 275 | 1.35 | 1.15-1.57 | 2E-04 | 1.42 | 1.19-1.69 | 8E-05 | |
|  | Women | BMI age 18-20 | 126,161 | 269 | 1.36 | 1.11-1.66 | 3E-03 | 1.19 | 0.95-1.50 | 0.14 |  | 124,308 | 238 | 1.36 | 1.10-1.68 | 4E-03 | 1.19 | 0.93-1.52 | 0.17 | |
|  | Women | Adult BMI | 126,161 | 269 | 1.26 | 1.11-1.43 | 3E-04 | 1.20 | 1.04-1.39 | 0.01 |  | 124,308 | 238 | 1.27 | 1.11-1.45 | 4E-04 | 1.21 | 1.04-1.41 | 0.01 | |
| **Pancreatic**  **cancer** | All | BMI age 18-20 | 185,361 | 689 | 1.12 | 0.97-1.30 | 0.11 | 1.06 | 0.91-1.25 | 0.46 |  | 182,315 | 647 | 1.14 | 0.98-1.32 | 0.09 | 1.07 | 0.90-1.26 | 0.45 | |
|  | All | Adult BMI | 185,361 | 689 | 1.11 | 1.01-1.21 | 0.03 | 1.09 | 0.98-1.21 | 0.11 |  | 182,315 | 647 | 1.12 | 1.02-1.23 | 0.02 | 1.10 | 0.99-1.22 | 0.08 | |
|  | All | BMI age 18-20*Sex | 185,361 | 689 | 1.12 | 0.84-1.50 | 0.42 | 1.08 | 0.79-1.49 | 0.63 |  | 182,315 | 647 | 1.12 | 0.84-1.51 | 0.44 | 1.09 | 0.79-1.52 | 0.60 | |
|  | All | Adult BMI * Sex | 185,361 | 689 | 1.10 | 0.91-1.33 | 0.34 | 1.07 | 0.86-1.32 | 0.54 |  | 182,315 | 647 | 1.08 | 0.89-1.32 | 0.43 | 1.05 | 0.84-1.31 | 0.66 | |
|  | Men | BMI age 18-20 | 59,200 | 311 | 1.20 | 0.96-1.49 | 0.11 | 1.11 | 0.87-1.42 | 0.41 |  | 58,007 | 287 | 1.21 | 0.96-1.52 | 0.10 | 1.12 | 0.87-1.45 | 0.38 | |
|  | Men | Adult BMI | 59,200 | 311 | 1.17 | 1.00-1.36 | 0.05 | 1.13 | 0.95-1.34 | 0.17 |  | 58,007 | 287 | 1.17 | 0.99-1.37 | 0.06 | 1.13 | 0.94-1.35 | 0.19 | |
|  | Women | BMI age 18-20 | 126,161 | 378 | 1.08 | 0.89-1.30 | 0.45 | 1.03 | 0.83-1.27 | 0.81 |  | 124,308 | 360 | 1.09 | 0.90-1.32 | 0.39 | 1.03 | 0.83-1.27 | 0.82 | |
|  | Women | Adult BMI | 126,161 | 378 | 1.08 | 0.96-1.21 | 0.22 | 1.07 | 0.94-1.22 | 0.31 |  | 124,308 | 360 | 1.10 | 0.97-1.23 | 0.13 | 1.09 | 0.95-1.24 | 0.21 | |
| **Lung**  **cancer** | All | BMI age 18-20 | 185,361 | 2,270 | 1.04 | 0.96-1.13 | 0.31 | 1.24 | 1.13-1.35 | 2E-06 |  | 182,315 | 2,104 | 1.04 | 0.96-1.14 | 0.31 | 1.24 | 1.13-1.36 | 6E-06 | |
|  | All | Adult BMI | 185,361 | 2,270 | 0.80 | 0.76-0.85 | 1E-13 | 0.76 | 0.71-0.81 | 0.00 |  | 182,315 | 2,104 | 0.81 | 0.76-0.86 | 5E-12 | 0.76 | 0.71-0.81 | 6E-16 | |
|  | All | BMI age 18-20*Sex | 185,361 | 2,270 | 1.14 | 0.97-1.34 | 0.11 | 1.19 | 1.00-1.42 | 0.05 |  | 182,315 | 2,104 | 1.14 | 0.96-1.35 | 0.14 | 1.21 | 1.00-1.45 | 0.05 | |
|  | All | Adult BMI * Sex | 185,361 | 2,270 | 0.97 | 0.86-1.09 | 0.61 | 0.91 | 0.80-1.04 | 0.15 |  | 182,315 | 2,104 | 0.95 | 0.84-1.07 | 0.38 | 0.88 | 0.77-1.01 | 0.07 | |
|  | Men | BMI age 18-20 | 59,200 | 1,068 | 1.08 | 0.96-1.22 | 0.22 | 1.33 | 1.16-1.52 | 4E-05 |  | 58,007 | 983 | 1.08 | 0.95-1.23 | 0.23 | 1.34 | 1.16-1.54 | 5E-05 | |
|  | Men | Adult BMI | 59,200 | 1,068 | 0.77 | 0.71-0.85 | 7E-08 | 0.71 | 0.64-0.78 | 5E-11 |  | 58,007 | 983 | 0.77 | 0.70-0.85 | 1E-07 | 0.70 | 0.63-0.78 | 1E-10 | |
|  | Women | BMI age 18-20 | 126,161 | 1,202 | 1.01 | 0.91-1.13 | 0.80 | 1.18 | 1.05-1.33 | 5E-03 |  | 124,308 | 1,121 | 1.02 | 0.91-1.14 | 0.77 | 1.17 | 1.04-1.33 | 0.01 | |
|  | Women | Adult BMI | 126,161 | 1,202 | 0.82 | 0.76-0.89 | 2E-07 | 0.79 | 0.73-0.85 | 5E-09 |  | 124,308 | 1,121 | 0.84 | 0.78-0.90 | 4E-06 | 0.80 | 0.74-0.87 | 1E-07 | |
| Squamous cell | All | BMI age 18-20 | 185,361 | 433 | 1.27 | 1.06-1.51 | 9E-03 | 1.55 | 1.29-1.87 | 4E-06 |  | 182,315 | 402 | 1.28 | 1.06-1.53 | 9E-03 | 1.56 | 1.29-1.90 | 6E-06 | |
|  | All | Adult BMI | 185,361 | 433 | 0.8 | 0.70-0.91 | 1E-03 | 0.70 | 0.60-0.81 | 2E-06 |  | 182,315 | 402 | 0.80 | 0.70-0.92 | 2E-03 | 0.70 | 0.60-0.82 | 5E-06 | |
|  | All | BMI age 18-20*Sex | 185,361 | 433 | 1.30 | 0.91-1.87 | 0.15 | 1.17 | 0.80-1.72 | 0.41 |  | 182,315 | 402 | 1.38 | 0.95-2.00 | 0.09 | 1.26 | 0.85-1.88 | 0.25 | |
|  | All | Adult BMI * Sex | 185,361 | 433 | 1.28 | 0.97-1.69 | 0.08 | 1.19 | 0.88-1.61 | 0.26 |  | 182,315 | 402 | 1.26 | 0.95-1.68 | 0.11 | 1.14 | 0.84-1.56 | 0.39 | |
|  | Men | BMI age 18-20 | 59,200 | 262 | 1.37 | 1.09-1.73 | 8E-03 | 1.62 | 1.26-2.09 | 2E-04 |  | 58,007 | 238 | 1.42 | 1.12-1.81 | 4E-03 | 1.69 | 1.30-2.21 | 9E-05 | |
|  | Men | Adult BMI | 59,200 | 262 | 0.88 | 0.73-1.05 | 0.16 | 0.75 | 0.61-0.92 | 5E-03 |  | 58,007 | 238 | 0.88 | 0.72-1.06 | 0.18 | 0.74 | 0.60-0.91 | 5E-03 | |
|  | Women | BMI age 18-20 | 126,161 | 171 | 1.14 | 0.86-1.50 | 0.37 | 1.45 | 1.09-1.94 | 0.01 |  | 124,308 | 164 | 1.11 | 0.83-1.49 | 0.47 | 1.41 | 1.05-1.90 | 0.02 | |
|  | Women | Adult BMI | 126,161 | 171 | 0.71 | 0.58-0.88 | 1E-03 | 0.65 | 0.52-0.81 | 1E-04 |  | 124,308 | 164 | 0.72 | 0.58-0.89 | 2E-03 | 0.66 | 0.53-0.83 | 3E-04 | |
| Adeno | All | BMI age 18-20 | 185,361 | 806 | 0.94 | 0.82-1.08 | 0.39 | 1.24 | 1.06-1.44 | 6E-03 |  | 182,315 | 752 | 0.94 | 0.81-1.09 | 0.39 | 1.23 | 1.06-1.44 | 8E-03 | |
|  | All | Adult BMI | 185,361 | 806 | 0.67 | 0.61-0.75 | 2E-14 | 0.64 | 0.57-0.71 | 8E-16 |  | 182,315 | 752 | 0.68 | 0.61-0.75 | 2E-13 | 0.64 | 0.57-0.71 | 8E-15 | |
|  | All | BMI age 18-20*Sex | 185,361 | 806 | 1.08 | 0.81-1.44 | 0.62 | 1.01 | 0.74-1.39 | 0.93 |  | 182,315 | 752 | 1.03 | 0.76-1.38 | 0.87 | 0.96 | 0.70-1.33 | 0.83 | |
|  | All | Adult BMI * Sex | 185,361 | 806 | 1.11 | 0.90-1.37 | 0.33 | 1.09 | 0.87-1.38 | 0.46 |  | 182,315 | 752 | 1.10 | 0.88-1.37 | 0.39 | 1.10 | 0.86-1.40 | 0.44 | |
|  | Men | BMI age 18-20 | 59,200 | 321 | 0.97 | 0.77-1.23 | 0.82 | 1.24 | 0.96-1.60 | 0.10 |  | 58,007 | 300 | 0.94 | 0.74-1.20 | 0.62 | 1.19 | 0.91-1.55 | 0.20 | |
|  | Men | Adult BMI | 59,200 | 321 | 0.72 | 0.61-0.85 | 1E-04 | 0.67 | 0.56-0.81 | 4E-05 |  | 58,007 | 300 | 0.72 | 0.60-0.86 | 2E-04 | 0.68 | 0.56-0.83 | 1E-04 | |
|  | Women | BMI age 18-20 | 126,161 | 485 | 0.92 | 0.77-1.10 | 0.37 | 1.23 | 1.02-1.49 | 0.03 |  | 124,308 | 452 | 0.94 | 0.78-1.13 | 0.48 | 1.25 | 1.03-1.52 | 0.02 | |
|  | Women | Adult BMI | 126,161 | 485 | 0.65 | 0.57-0.74 | 3E-11 | 0.62 | 0.54-0.71 | 3E-12 |  | 124,308 | 452 | 0.65 | 0.57-0.75 | 2E-10 | 0.62 | 0.54-0.71 | 1E-11 | |
| Small cell | All | BMI age 18-20 | 185,361 | 293 | 1.35 | 1.10-1.65 | 4E-03 | 1.31 | 1.04-1.65 | 0.02 |  | 182,315 | 268 | 1.40 | 1.14-1.72 | 1E-03 | 1.36 | 1.08-1.72 | 0.01 | |
|  | All | Adult BMI | 185,361 | 293 | 1.13 | 0.98-1.30 | 0.08 | 1.05 | 0.89-1.22 | 0.58 |  | 182,315 | 268 | 1.15 | 0.99-1.33 | 0.06 | 1.05 | 0.89-1.23 | 0.59 | |
|  | All | BMI age 18-20*Sex | 185,361 | 293 | 0.89 | 0.59-1.35 | 0.60 | 0.87 | 0.55-1.39 | 0.56 |  | 182,315 | 268 | 0.95 | 0.62-1.45 | 0.80 | 0.95 | 0.59-1.53 | 0.83 | |
|  | All | Adult BMI * Sex | 185,361 | 293 | 1.01 | 0.75-1.35 | 0.97 | 1.04 | 0.75-1.44 | 0.80 |  | 182,315 | 268 | 0.99 | 0.73-1.34 | 0.96 | 1.00 | 0.71-1.40 | 1.00 | |
|  | Men | BMI age 18-20 | 59,200 | 134 | 1.18 | 0.84-1.65 | 0.34 | 1.14 | 0.78-1.65 | 0.51 |  | 58,007 | 124 | 1.28 | 0.91-1.81 | 0.15 | 1.26 | 0.86-1.85 | 0.24 | |
|  | Men | Adult BMI | 59,200 | 134 | 1.10 | 0.87-1.40 | 0.42 | 1.06 | 0.81-1.38 | 0.66 |  | 58,007 | 124 | 1.11 | 0.87-1.42 | 0.40 | 1.03 | 0.78-1.36 | 0.82 | |
|  | Women | BMI age 18-20 | 126,161 | 159 | 1.46 | 1.14-1.86 | 2E-03 | 1.43 | 1.08-1.88 | 0.01 |  | 124,308 | 144 | 1.48 | 1.15-1.90 | 2E-03 | 1.43 | 1.07-1.91 | 0.01 | |
|  | Women | Adult BMI | 126,161 | 159 | 1.15 | 0.97-1.36 | 0.12 | 1.04 | 0.85-1.25 | 0.72 |  | 124,308 | 144 | 1.17 | 0.98-1.40 | 0.09 | 1.05 | 0.86-1.28 | 0.62 | |
| **Ovarian**  **cancer** | Women | BMI age 18-20 | 126,161 | 723 | 1.06 | 0.92-1.21 | 0.43 | 1.02 | 0.87-1.19 | 0.82 |  | 124,308 | 649 | 1.09 | 0.95-1.26 | 0.22 | 1.04 | 0.88-1.22 | 0.68 | |
|  | Women | Adult BMI | 126,161 | 723 | 1.06 | 0.97-1.15 | 0.19 | 1.05 | 0.96-1.16 | 0.28 |  | 124,308 | 649 | 1.09 | 1.00-1.19 | 0.06 | 1.08 | 0.98-1.19 | 0.14 | |
| **Endometrial**  **cancer** | Women | BMI age 18-20 | 126,161 | 914 | 1.19 | 1.06-1.34 | 3E-03 | 0.84 | 0.73-0.96 | 9E-03 |  | 124,308 | 831 | 1.19 | 1.05-1.35 | 5E-03 | 0.82 | 0.71-0.94 | 6E-03 |  |
|  | Women | Adult BMI | 126,161 | 914 | 1.47 | 1.38-1.56 | 0.00 | 1.54 | 1.44-1.65 | 0.00 |  | 124,308 | 831 | 1.49 | 1.40-1.59 | 0.00 | 1.57 | 1.46-1.69 | 0.00 |  |
| Endometrioid | Women | BMI age 18-20 | 126,161 | 507 | 1.27 | 1.09-1.48 | 2E-03 | 0.87 | 0.73-1.04 | 0.12 |  | 124,308 | 462 | 1.23 | 1.04-1.44 | 0.01 | 0.83 | 0.68-1.00 | 0.05 |  |
|  | Women | Adult BMI | 126,161 | 507 | 1.52 | 1.41-1.65 | 0.00 | 1.58 | 1.44-1.74 | 0.00 |  | 124,308 | 462 | 1.52 | 1.40-1.65 | 0.00 | 1.60 | 1.45-1.77 | 0.00 |  |
| Non-endometrioid | Women | BMI age 18-20 | 126,161 | 407 | 1.09 | 0.91-1.31 | 0.35 | 0.79 | 0.65-0.98 | 0.03 |  | 124,308 | 369 | 1.14 | 0.95-1.38 | 0.16 | 0.81 | 0.65-1.00 | 0.05 | |
|  | Women | Adult BMI | 126,161 | 407 | 1.40 | 1.28-1.54 | 2E-12 | 1.49 | 1.34-1.66 | 3E-13 |  | 124,308 | 369 | 1.45 | 1.32-1.60 | 4E-14 | 1.53 | 1.37-1.71 | 3E-14 | |

^a^ Hazard ratios (HRs) and 95% confidence intervals for BMI reflect a 5 kg/m2 increase. HRs are adjusted for age, cohort and sex.

^b^ Hazard ratios (HRs) and 95% confidence intervals for BMI reflect a 5 kg/m2 increase. HRs are adjusted for age, cohort, sex and the other BMI exposure.

^c^ Subsample of participants with non-missing weight at age 18-20

^d^ Women are the reference group in the models with interaction between BMI and sex

eTable 4. Cox regression results after adjustment for cancer risk factors at recruitment.

|  |  |  |  | **Exposure-specific**  **models^a^** | | | **Mutually-adjusted**  **model^b^** | | |
| --- | --- | --- | --- | --- | --- | --- | --- | --- | --- |
| **Outcome** | **Exposure^d^** | **N** | **N cancer** | **HR** | **95%CI** | ***P*** | **HR** | **95%CI** | ***P*** |
| **Colorectal**  **cancer** | BMI at age 18-20 | 105,705 | 1,852 | 1.04 | 0.96-1.14 | 0.33 | 0.94 | 0.85-1.04 | 0.22 |
|  | Adult BMI | 105,705 | 1,852 | 1.15 | 1.09-1.21 | 1E-06 | 1.17 | 1.10-1.24 | 1E-06 |
| **Kidney**  **cancer** | BMI at age 18-20 | 105,705 | 364 | 1.13 | 0.94-1.38 | 0.20 | 0.95 | 0.77-1.18 | 0.67 |
|  | Adult BMI | 105,705 | 364 | 1.28 | 1.13-1.44 | 8E-05 | 1.30 | 1.13-1.48 | 2E-04 |
| **Pancreatic**  **cancer** | BMI at age 18-20 | 105,705 | 415 | 1.01 | 0.83-1.22 | 0.95 | 0.91 | 0.74-1.13 | 0.40 |
|  | Adult BMI | 105,705 | 415 | 1.13 | 1.00-1.27 | 0.05 | 1.15 | 1.01-1.32 | 0.03 |
| **Lung**  **cancer** | BMI at age 18-20 | 105,705 | 1,360 | 0.93 | 0.84-1.03 | 0.18 | 1.01 | 0.90-1.13 | 0.85 |
|  | Adult BMI | 105,705 | 1,360 | 0.87 | 0.81-0.94 | 2E-04 | 0.87 | 0.81-0.94 | 1E-03 |
| Squamous cell  carcinoma | BMI at age 18-20 | 105,705 | 254 | 1.02 | 0.81-1.29 | 0.86 | 1.14 | 0.89-1.47 | 0.29 |
|  | Adult BMI | 105,705 | 254 | 0.85 | 0.71-1.00 | 0.05 | 0.81 | 0.68-0.98 | 0.03 |
| Adenocarcinoma | BMI at age 18-20 | 105,705 | 499 | 0.89 | 0.75-1.06 | 0.18 | 1.09 | 0.91-1.31 | 0.37 |
|  | Adult BMI | 105,705 | 499 | 0.72 | 0.63-0.81 | 1E-07 | 0.70 | 0.61-0.80 | 2E-07 |
| Small cell  lung cancer | BMI at age 18-20 | 105,705 | 214 | 1.10 | 0.87-1.39 | 0.42 | 0.99 | 0.76-1.28 | 0.94 |
|  | Adult BMI | 105,705 | 214 | 1.19 | 1.01-1.39 | 0.03 | 1.19 | 1.00-1.41 | 0.05 |
| **Ovarian**  **cancer** | BMI at age 18-20 | 63,963 | 392 | 1.05 | 0.87-1.26 | 0.64 | 0.98 | 0.79-1.21 | 0.86 |
|  | Adult BMI | 63,963 | 392 | 1.09 | 0.97-1.22 | 0.16 | 1.09 | 0.96-1.24 | 0.18 |
| **Endometrial**  **cancer** | BMI at age 18-20 | 63,963 | 476 | 1.14 | 0.97-1.35 | 0.11 | 0.82 | 0.68-1.00 | 0.05 |
|  | Adult BMI | 63,963 | 476 | 1.44 | 1.32-1.57 | 3E-16 | 1.51 | 1.37-1.67 | 2E-16 |
| Endometrioid | BMI at age 18-20 | 63,963 | 289 | 1.25 | 1.02-1.53 | 0.03 | 0.91 | 0.72-1.16 | 0.46 |
|  | Adult BMI | 63,963 | 289 | 1.44 | 1.28-1.61 | 2E-10 | 1.47 | 1.30-1.68 | 3E-09 |
| Non-  endometrioid | BMI at age 18-20 | 63,963 | 187 | 0.99 | 0.75-1.30 | 0.93 | 0.70 | 0.51-0.95 | 0.02 |
|  | Adult BMI | 63,963 | 187 | 1.43 | 1.25-1.64 | 3E-07 | 1.56 | 1.34-1.81 | 9E-09 |

^a^ Hazard ratio (HRs) and 95% confidence intervals for BMI reflect a 5 kg/m2 increase. HRs are adjusted for age, cohort, sex, smoking history before recruitment (lifetime cigarettes/day, smoking duration, current smoking, former smoking, time since quitting, interaction between former smoking and time since quitting), average alcohol intake, physical activity score, highest achieved education and age at menarche (for female cancers).

^b^ Hazard ratios (HRs) and 95% confidence intervals for BMI reflect a 5 kg/m2 increase. HRs are adjusted for age, cohort, sex, smoking history before recruitment (lifetime cigarettes/day, smoking duration, current smoking, former smoking, time since quitting, interaction between former smoking and time since quitting), average alcohol intake, physical activity score, highest achieved education, age at menarche (for female cancers) and the other BMI exposure.

^c^ Subsample of participants with non-missing weight at age 18-20

# eFigures

##
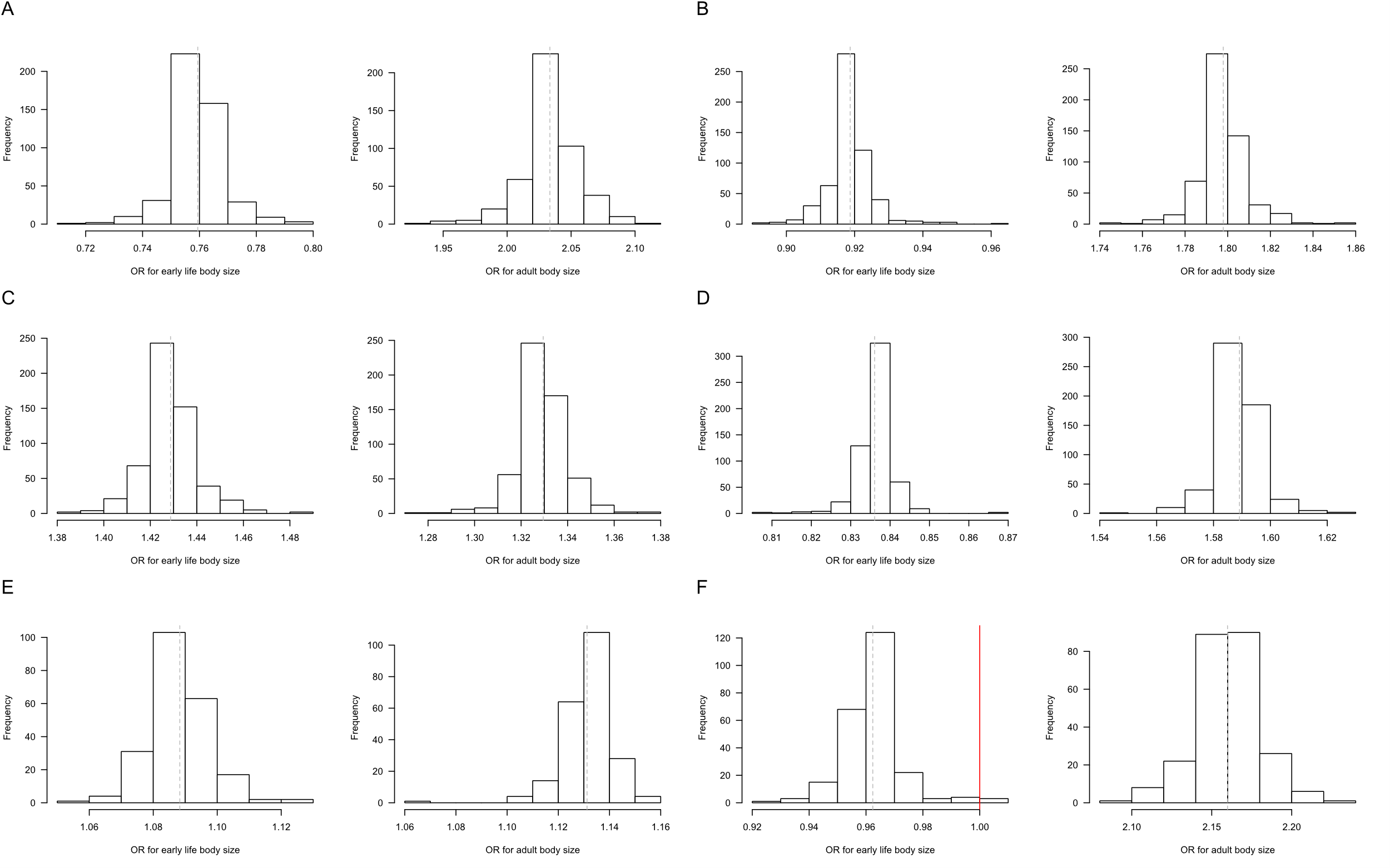
**eFigure 1. Leave-one-out multivariable Mendelian randomization using the inverse-variance weighted method for cancer of the colorectum (A), kidney (B), pancreas (C), lung (D), ovary (E), and endometrium (F).** Dashed lines show the results in the main analysis that includes all SNPs.

eFigure 2. Influential SNPs in univariable (UV) and multivariable (MV) Mendelian randomization for cancer of the colorectum (A), kidney (B), pancreas (C), lung (D), ovary (E), and endometrium (F). The cut-off for influential values based on Cook’s distance was 4/number of SNPs in the model. The influential SNPs are sorted by the number of pleiotropic traits (annotation).


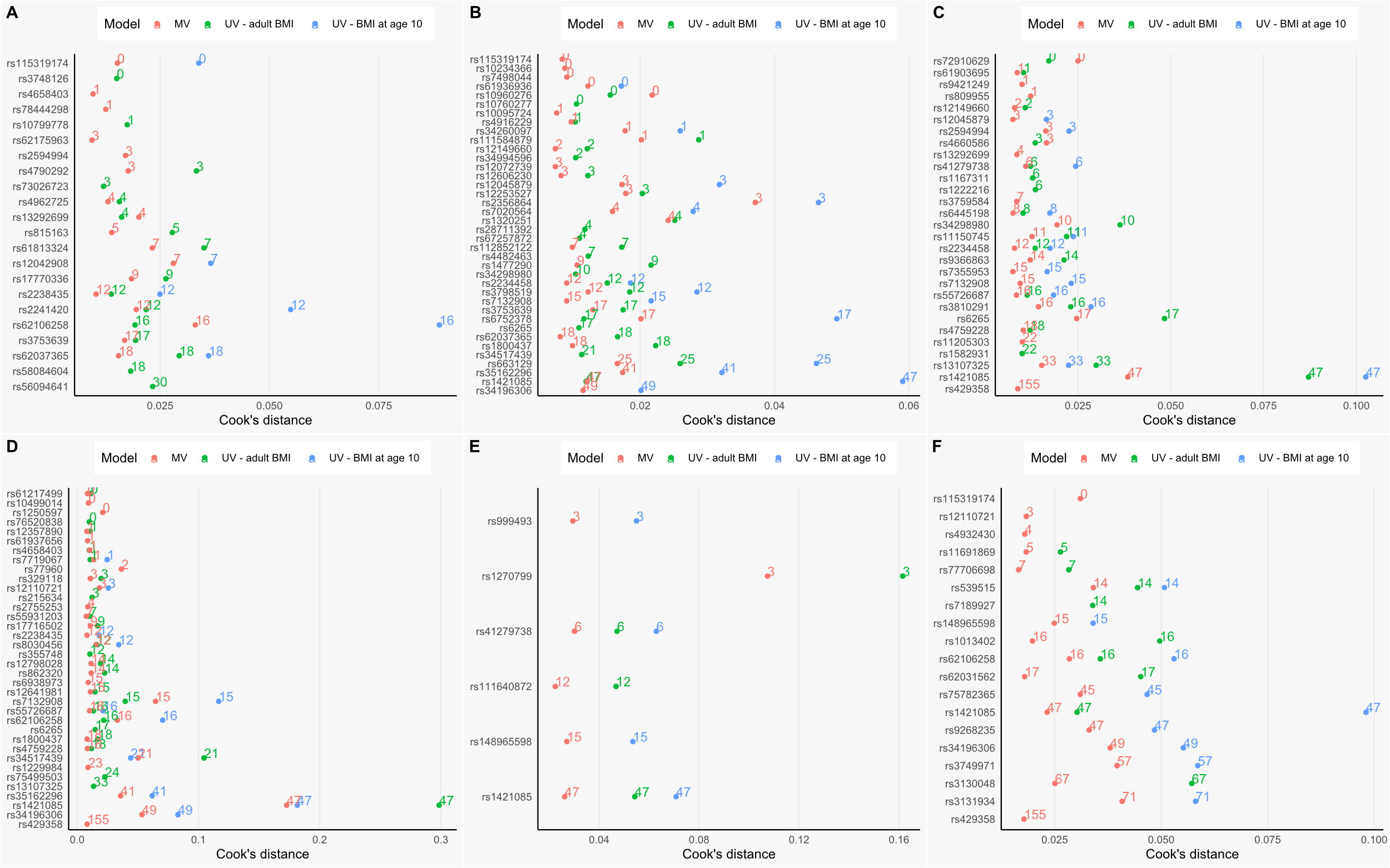


eFigure 3. Ridge regression estimates for body size and risk of cancer of the colorectum (A), kidney (B), pancreas (C), lung (D), ovary (E), and endometrium (F) and for body mass index in the EPIC cohort and risk of cancer of the colorectum (F), kidney (G), pancreas (H), lung (I), ovary (J), and endometrium (K). Dashed lines are at λ value from cross-validation and confidence bands refer to 95% confidence levels.


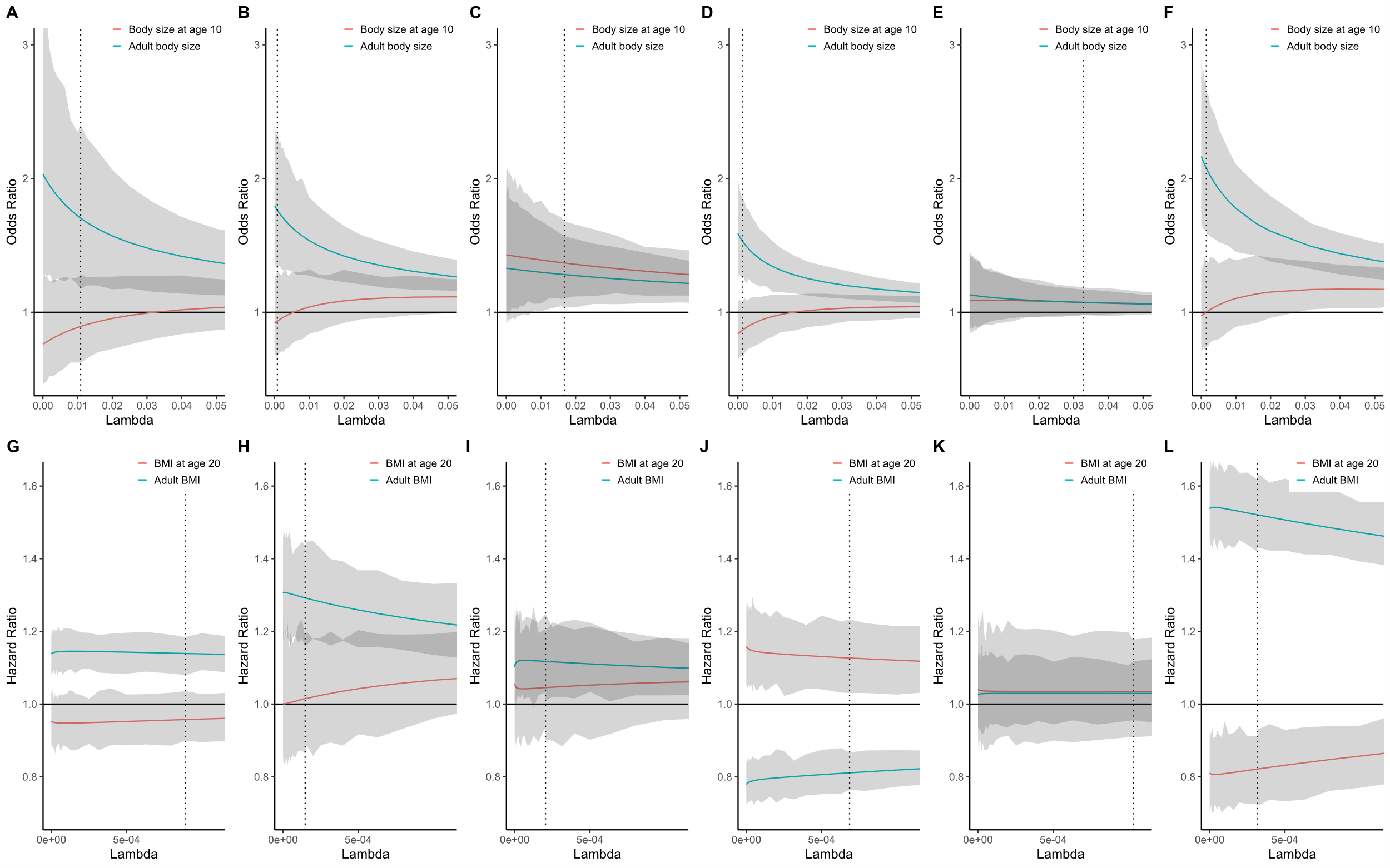

Supplement: djac061_Supplementary_Data [file djac061_supplementary_data.docx]
